# Data quality biases normative models derived from fetal brain MRI

**DOI:** 10.1162/IMAG.a.1337

**Published:** 2026-08-14

**Authors:** Thomas Sanchez, Angeline Mihailov, Gerard Martí-Juan, Nadine Girard, Aurélie Manchon, Mathieu Milh, Elisenda Eixarch, Vincent Dunet, Mériam Koob, Léo Pomar, Joanna Sichitiu, Miguel A. González Ballester, Oscar Camara, Gemma Piella, Meritxell Bach Cuadra, Guillaume Auzias

**Affiliations:** CIBM Center for Biomedical Imaging, Lausanne, Switzerland; Department of Medical Radiology, Lausanne University Hospital and University of Lausanne, Lausanne, Switzerland; Institut de Neurosciences de la Timone, CNRS, Aix-Marseille Université, Marseille, France; BCN MedTech, Department of Engineering, Universitat Pompeu Fabra, Barcelona, Spain; APHM, Service de Neuroradiologie Diagnostique et Interventionnelle, Hôpital de la Timone, Aix-Marseille University, Marseille, France; APHM, Service de Neurologie Pédiatrique, Hôpital de la Timone, Aix-Marseille University, Marseille, France; Aix Marseille University, Inserm, Marseille, France; BCNatal | Fetal Medicine Research Center (Hospital Clínic and Hospital Sant Joan de Déu, Universitat de Barcelona), Barcelona, Spain; Institut d’Investigacions Biomèdiques August Pi i Sunyer (IDIBAPS), Barcelona, Spain; Centre for Biomedical Research on Rare Diseases (CIBERER), Barcelona, Spain; Department Woman-Mother-Child, Lausanne University Hospital and University of Lausanne, Lausanne, Switzerland; School of Health Sciences (HESAV), HES-SO, University of Applied Sciences and Arts Western Switzerland, Lausanne, Switzerland; ICREA, Barcelona, Spain

**Keywords:** fetal, brain MRI, normative modeling, image artifacts, quality control

## Abstract

Normative modeling is increasingly used to characterize typical growth trajectories and identify atypical neurodevelopment, including early brain development using magnetic resonance imaging (MRI) acquired before birth. Recent work has emphasized the importance of large sample sizes for accurate and robust centile estimation. In this study, we investigate how image quality influences fetal brain normative models, a critical factor in this context where MRI is acquired on a moving fetus *in utero*. Using a multi-centric cohort of 635 fetal MRI scans, we applied a standardized visual quality control (QC) protocol with continuous quality ratings. We fit normative models for multiple brain structures under progressively relaxed QC stringency, and quantified the deviations in centile estimates relative to a high-quality reference subgroup. Our results showed that including lower-quality data systematically biased normative centiles, with the strongest effects observed in the outer centiles, particularly the lower tail (1st–10th). Bias increased progressively as QC stringency was relaxed and could not be attributed solely to the number of scans used to fit the models. Quality-induced bias was structure dependent, and often not visually apparent at the segmentation level. These findings highlight that image quality is an important source of bias in normative fetal brain modeling, and that increasing sample size at the expense of quality may systematically affect centile estimates, potentially jeopardizing the utility of the model.

## 1 Introduction

Accurate and robust estimation of normative trajectories of brain development is crucial for detecting atypical neurodevelopmental processes and potential neurological disorders (Bethlehem et al., 2022; Fraza et al., 2025; Kyriakopoulou et al., 2017; Rutherford et al., 2022). Structural magnetic resonance imaging (MRI) has become increasingly used to study the developing brain, offering a non-invasive means to characterize typical and atypical neurodevelopmental trajectories throughout gestation and into early postnatal life (Bethlehem et al., 2022; Ji et al., 2025; Kyriakopoulou et al., 2017; Machado-Rivas et al., 2022; Mihailov et al., 2025). However, the reliability and validity of measures derived from fetal MRI directly depend on the quality of the acquired images, which can be affected by fetal motion, maternal physiological noise, and scanner-related artifacts. Rigorous quality control (QC) is particularly critical in fetal imaging, where rapid anatomical changes throughout gestational age interact with complex reconstruction and segmentation pipelines, potentially introducing subtle and structure-specific artifacts (Sanchez et al., 2024).

While quality control (QC) protocols are now well established in adult brain MRI (Alfaro-Almagro et al., 2018; Backhausen et al., 2016; Esteban et al., 2017; Kim et al., 2025; Monereo-Sánchez et al., 2021), this is not the case in fetal brain imaging. In this field, qualitative visual inspection of each scan remains common practice to exclude images with severe artifacts or poor contrast (Ducharme et al., 2016; Namburete et al., 2023; Rosen et al., 2018). Despite the major potential impact of QC on the reported results, the description of the operational criteria is often insufficient to enable other groups to reproduce the full procedure, which impedes both the comparison and reproducibility of results across studies. In addition, while most studies report excluding low-quality data, the impact of varying degrees of data quality—beyond binary inclusion or exclusion—on normative modeling results is rarely investigated. More specifically, the impact of sub-optimal data quality on the estimation of fetal brain normative trajectories and centiles has not been investigated. In this work, we address the following questions:

1. How does the inclusion of sub-optimal quality MRI scans bias the estimation of median and outer centiles in fetal brain normative models?
2. To what extent is this bias driven by image quality itself, rather than changes in effective sample size induced by quality-based data exclusion?

To answer these questions, we investigate the effect of varying visual QC stringency on normative centile predictions for multiple fetal brain structures, using a large multi-centric cohort of 635 fetal MRI scans. Leveraging a recently released standardized visual QC protocol (Bach Cuadra et al., 2025; Sanchez et al., 2024) that provides continuous image-level quality ratings, we define nested QC subgroups of increasing stringency and quantify the resulting centile bias relative to a high-quality reference subgroup. Because site harmonization is now standard practice in multi-centric normative modeling, we additionally examine how data quality interacts with harmonization, as the latter may absorb quality-related variance and partially mask its effects. We further disentangle the respective contributions of data quality and sample size by means of a weighted bootstrapping strategy that allows independent control over sample size and average data quality. Together, these analyses aim to characterize how data quality systematically influences fetal brain normative models, beyond simple inclusion–exclusion decisions, and to assess the extent to which increasing sample size can compensate for lower data quality.

## 2 Methods

### 2.1 Data

#### 2.1.1 Cohorts and demographics

We combined MRI datasets acquired in clinical routine and in research settings. To establish a healthy fetal cohort, a multidisciplinary team, including specialists in neuroradiology, obstetrics, and pediatric neurology, formalized a list of exclusion criteria to define standardized clinical and anatomical normality across sites. The detailed inclusion and exclusion criteria are provided in Supplementary Section S1 (Supplementary Table S1). Applying these criteria led to a cohort of 635 fetal scans (261:326:48 (F:M:unknown), age range between 20.85 and 38.86 weeks of gestational age). Table 1 reports the demographic information of the dataset included in this study, after application of the exclusion criteria.

**Table 1.** Summary of fetal MRI datasets.

Dataset	N	Sex ratio (F:M:Unknown)	Age (Weeks)	Scanner Manufacturer	Scanner repartition (T; Model name)	${\text{n}}_{\text{stacks}}$ (mean±SD) [min, max]
dHCP	292	133:157:2	21–38	Philips	292 (3 T; Achieva) 0 (1.5 T)	4.9 ± 3.3 [2, 31]
MarsFet	216	83:90:43	21–38	Siemens	114 (3 T; Skyra) 102 (1.5 T; SymphonyTim)	3.9 ± 1.4 [3, 9]
BCNatal	91	33:55:3	26–39	Siemens	59 (3 T; TrioTim) 32 (1.5 T; Aera)	6.9 ± 4.2 [3, 21]
CHUV	36	12:24:0	21–36	Siemens	3 (3 T; Skyra) 33 (1.5 T; MAGNETOM Sola)	7.2 ± 2.9 [3, 16]

##### 2.1.1.1 Clinical dataset

The clinical fetal MRI data were acquired with different Siemens Healthineers scanners (Erlangen, Germany) at 1.5 Tesla (T) or 3 T across hospitals. The fetal brain MRI protocol included T2w HASTE (Half-Fourier Acquisition Single-shot Turbo spin Echo imaging) sequences acquired in at least three orthogonal directions (axial, coronal, sagittal). The number of stacks acquired in each site is reported in Table 1, and we provide below the information that is specific to each dataset.

1. **MarsFet.** The MarsFet fetal dataset was assembled retrospectively from MRI examinations performed during routine prenatal clinical care at La Timone Hospital in Marseille, France, between 2008 and 2021. Ethical approval was obtained from the institutional review board (N°2022-04-14-003). The data were acquired on two Siemens scanners (Skyra 3 T and SymphonyTim 1.5 T) with at least three stacks acquired in orthogonal orientations. Spatial resolution was between 0.6 and 0.7 mm in-plane and slice thickness between 3 and 3.6 mm. After applying the predefined exclusion criteria, 216 fetal scans (1 scan per subject) were retained for analysis.
2. **BCNatal.** The BCNatal dataset was retrospectively assembled from fetal MRI scans of uncomplicated pregnancies acquired at Hospital Clínic Barcelona and Hospital Sant Joan de Déu, Spain, grouped around 28 and 34 weeks of gestational age (GA), where MRI examinations are typically performed. Ethical approval was obtained from the institutional review board (HCB/2022/0533). The data were acquired on two Siemens scanners (TrioTim 3 T and Aera 1.5 T), with a minimum of four stacks per scanning session, including at least one in each orthogonal plane. Spatial resolution was $0.51\times 0.51\times 3.5{\text{mm}}^{3}$ at 3 T and 0.55×0.55× $3.6{\text{mm}}^{3}$ at 1.5 T. After applying the predefined exclusion criteria, 91 fetal scans (1 scan per subject) were included in the analysis.
3. **CHUV.** The CHUV dataset was assembled retrospectively from MRI examinations performed during standard prenatal clinical care between 2013 and 2024 at the Lausanne University Hospital, Switzerland. Ethical approval was obtained from the institutional review board (CER-VD 2021-00124). The data were acquired on four Siemens scanners (Skyra and Skyra_fit at 3 T, and Aera and MAGNETOM Sola at 1.5 T), with a minimum of six stacks (at least two for each orthogonal plane). Spatial resolution was $0.55\times 0.55\times 3{\text{mm}}^{3}$ at 3 T and $1.1\times 1.1\times 3.3{\text{mm}}^{3}$ at 1.5 T. After applying the predefined exclusion criteria, 36 fetal scans (1 scan per subject) were retained for analysis.

##### 2.1.1.2 Research dataset. Fetal dHCP.

We also included the research dataset released by developing Human Connectome Project (dHCP)^1^ This dataset consists of 297 MRI sessions from 273 fetuses. As detailed in Price et al. (2019), MRI data were acquired on a 3 T Philips Achieva scanner. Structural T2w data were acquired from six uniquely oriented stacks centered on the fetal brain using a zoomed multiband single-shot TSE sequence, at an in-plane resolution of 1.1 mm and slice thickness of 2.2 mm. After application of the exclusion criteria, this dataset resulted in a final sample of 292 fetal scans.

### 2.2 Data processing

**Fig. 1.**
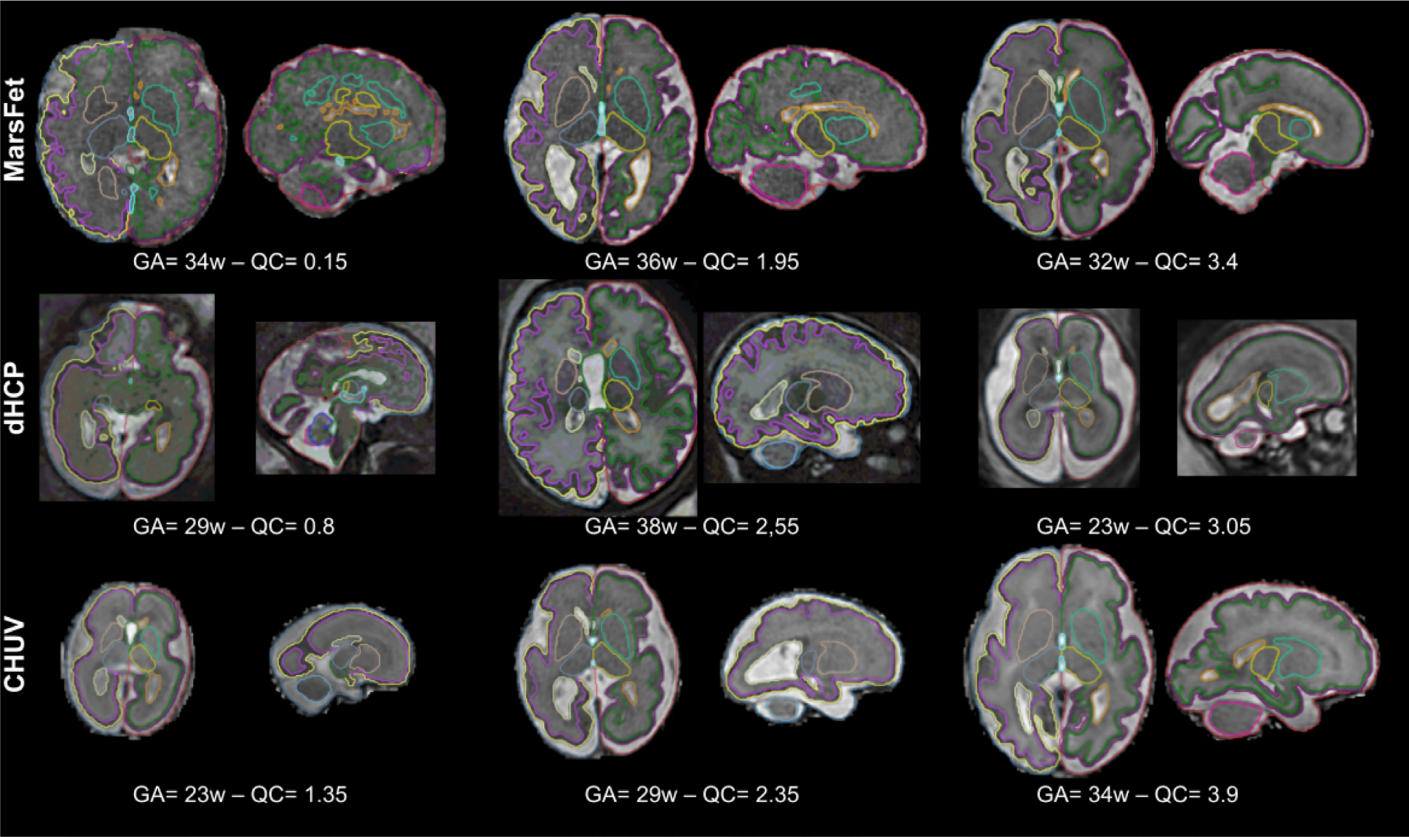
Examples of data from the different cohorts, with different gestational ages and different global QC ratings. Overlaid on each example are segmentations from BOUNTI, before aggregation into the eight categories of interest. Note that two slices are not sufficient to extensively reflect the quality of a given scan, as the rating is based on inspection of *all* slices, and artifacts often lie at the extremes. We refer the reader to Bach Cuadra et al. (2025) for additional examples.

To ensure reproducible processing across the datasets, we relied on Fetpype, an open source image processing pipeline designed to standardize the pre-processing, reconstruction, and segmentation of fetal brain MRI (Sanchez et al., 2026)^2^. An example of the processed data is available in Figure 1.

#### 2.2.1 Super-resolution reconstruction

Fetal brain MRI acquisitions are typically acquired using multiple orientations with high in-plane resolution (voxel size below 1 mm) and thick slices (usually 3–4 mm), which need to be combined into a single isotropic high-resolution volume (typically 0.5–0.8 mm resolution). For the fetal dHCP, we used the released 3D reconstructed volumes at 0.5 mm isotropic voxel resolution obtained using the method described in Cordero-Grande et al. (2022) and Edwards et al. (2022). For the clinical datasets, we reconstructed the 3D high-resolution volume using state-of-the art super-resolution reconstruction (SRR) methods integrated in Fetpype. The preprocessing and reconstruction in Fetpype consist of the following steps: brain extraction using Fetal-BET (Faghihpirayesh et al., 2024), non-local means denoising (Manjón et al., 2008), and N4 bias-field correction (Tustison et al., 2010), 3D volume reconstruction using NeSVoR (v.0.5.0) (J. Xu et al., 2023), including all available HASTE stacks to reconstruct each scan. Data were reconstructed at 0.5 mm isotropic voxel resolution for MarsFet and BCNatal, and 0.8 mm isotropic voxel resolution at CHUV. Several SRR methods are available in Fetpype. We evaluated alternative SRR techniques on similar data in previous work (Sanchez et al., 2025) and observed minimal impact on the volumetry measures. We used NeSVoR in this study because it runs faster than the alternative approaches. Although dHCP and clinical data were reconstructed using different SRR methods, previous work reported that volumetric measurements are consistent across SRR methods for a given segmentation method (Mihailov et al., 2025; Sanchez et al., 2025). We report in Section S7 of Supplementary Materials additional analyses in which we confirm that our results and conclusions remain unchanged dHCP dataset (Supplementary FIgs. S7 and S8).

#### 2.2.2 Segmentation

All data were then segmented using BOUNTI (Uus, Kyriakopoulou, Makropoulos, et al., 2023), which is integrated in Fetpype. BOUNTI’s segmentation features 19 different brain regions. For both hemispheres, it segments extra-cerebral CSF (**eCSF**), cortical gray matter (**cGM**), white matter (**WM**), lateral ventricles (**LV**), cerebellum, basal ganglia (**BG**), and thalamus. In addition, it also segments the brainstem, the cavum septum pellucidum, the cerebellar vermis, as well as the third and fourth ventricles (Uus, Kyriakopoulou, Cordero-Grande, et al., 2023). We aggregated anatomical structures across hemispheres in order to avoid potential lateralization effects that would be difficult to interpret in relation to global data QC measures. Additionally, we excluded small structures that could be affected by local variations in image quality (cavum septum pellucidum, cerebellar vermis, as well as third and fourth ventricles). We, therefore, focus our analysis on eight anatomical structures aggregated across hemispheres: eCSF, cGM, WM, LV, cerebellum, BG, thalamus, and brainstem.

### 2.3 Standardized visual quality assessment

**Fig. 2.**
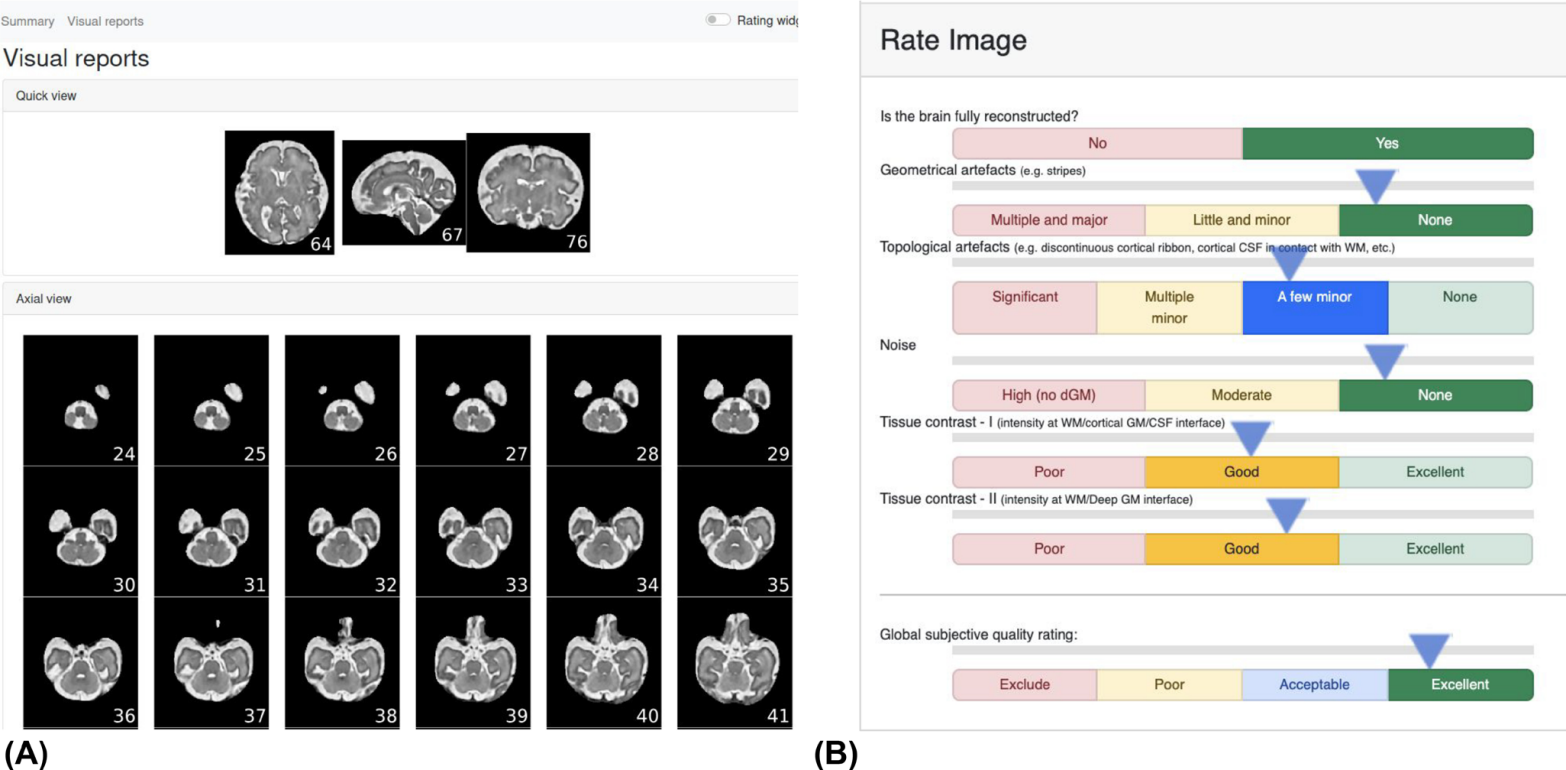
The QC interface used throughout the study. (A) The visualization interface first shows a quick view of each orientation in a given volume, before showing all slices in each orientation. (B) The rating interface, embedded in the HTML report, asks the rater to go through a guided assessment of the quality of the image, by rating six different criteria before giving a global subjective quality rating.

We then performed a visual quality assessment using FetMRQC, which is publicly available on GitHub. FetMRQC generates for each scan an HTML report that displays the data in a standardized layout with a rating interface, as illustrated in Figure 2. As detailed in Sanchez et al. (2024), the rater goes through a guided assessment of the quality of the image, according to the protocol available on Zenodo (Bach Cuadra et al., 2025). The guided assessment consists in scoring the following criteria:

- **Is the brain fully reconstructed?** [Binary]—Are there holes, or large parts of the brain missing?
- **Geometrical artifacts.** [Continuous] Did the SRR introduce any non-biological, textured artifact, such as stripes?
- **Topological artifacts.** [Continuous] Did the SRR introduce any discontinuity in the cortical gray matter (cGM), or are some parts of the white matter (WM) directly touching the cortical cerebrospinal fluid (CSF)?
- **Noise.** [Continuous] Is there a high level of noise in the image, potentially preventing us from seeing the deep gray matter (dGM) clearly?
- **Tissue intensity contrast – WM/cGM/cerebrospinal fluid.** [Continuous] Is the contrast sufficient? Do we see well the cGM?
- **Tissue intensity contrast – WM/dGM.** [Continuous] Is the contrast sufficient? Do we see well the sub-regions in the dGM?

All continuous scores range from 0 (very poor) to 4 (excellent), following the scales shown in Figure 2. After scoring the artifacts, the raters were asked to provide a final global subjective quality rating score for the image (on a continuous scale between 0 and 4), based on the previously assessed criteria. This global rating served as our quality reference throughout the study.

#### 2.3.1 Rater training

Three raters (A.M., G.M.J., T.S.) underwent a training session, where they were asked to rate 25 images from MarsFet, which led to an intra-class correlation coefficient ICC(A,1)=0.75 . *ICC*(*A*, 1) penalizes both random measurement errors and systematic differences between raters. It uses a two-way random-effects model with absolute agreement (*A*) rather than consistency (*C*), the latter discarding systematic inter-rater biases. The subscript 1 refers to single-rater reliability, as opposed to mean-of-raters reliability (*k* = 3 here), which quantifies the reliability of the mean score across all three raters (Koo & Li, 2016). Based on their rating, a discussion session was organized to review cases with strong disagreement. During the discussion, raters discussed extensively the cases they considered as ambiguous to understand the rationale for each rater’s choice and reach a consensus. Note that the agreement at the training session was already higher than the baseline agreement of ICC(A,1)=0.63 that we obtained at the training session of Sanchez et al. (2024).

After this discussion, each rater evaluated one part of the dataset (AM-rated MarsFet + dHCP, GMJ-rated BCNatal, and TS-rated CHUV), resulting in a detailed quality assessment for each of the 635 fetal MRI scans. We did not perform an additional inter-rater or intra-rater reliability, as a previous evaluation of the same protocol demonstrated a very high inter-rater reliability (ICC(A,1)=0.89 ) after the training and review session (Sanchez et al., 2024).

### 2.4 Analysis of the impact of image quality on normative models

#### 2.4.1 QC subgroups definition

**Fig. 3.**
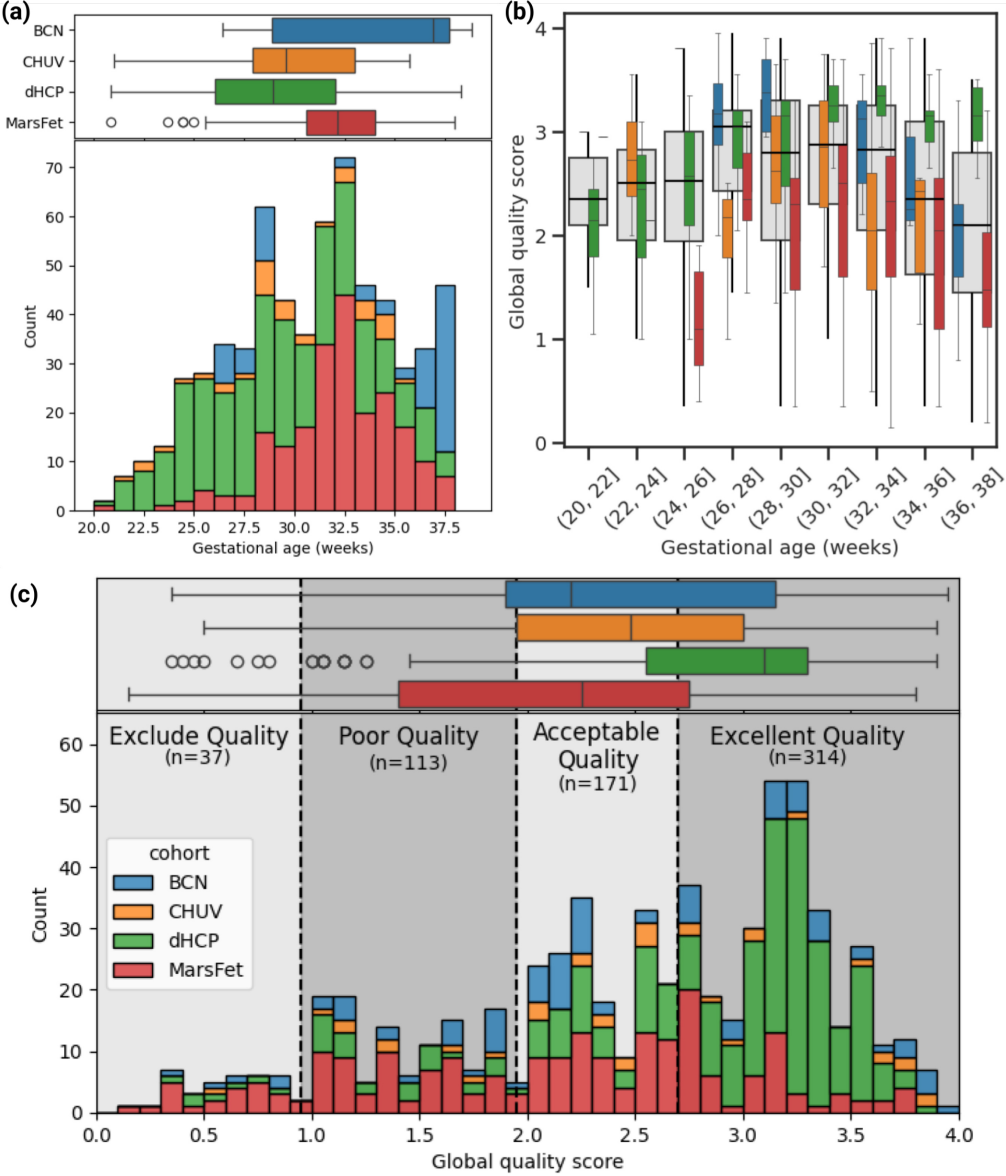
Quality and gestational age (GA) distribution of the data across the different cohorts. (a) Distribution of GA across datasets. (b) Distribution of global quality as a function of GA. (c) Distribution of the global quality score across the different datasets, with the excellent quality defined to include scans with a score  ≥2.7 .

The distribution of the image quality ratings across the four datasets is shown in Figure 3. Based on this distribution, we defined four quality groups, representing data with increasingly stringent QC criteria:

- All: quality scores ≥ 0, n = 635, QC = 2.5 ± 0.9
- Poor+: quality scores ≥ 1, n = 598, QC = 2.6 ± 0.7
- Accept+: quality scores ≥ 2, n = 485, QC = 2.9 ± 0.5
- GroundTruth: quality scores ≥ 2.7, n = 314, QC = 3.2 ± 0.3.

The threshold value of 2.7 for the GroundTruth subgroup was determined based on an experiment described in Supplementary Section S4 and Supplementary Figure S4. We started from the full cohort of 635 scans, which were ranked according to QC score. We then iteratively removed the lowest-quality data in batches of 10, generating a series of subgroups with progressively higher data quality and decreasing sample size. For each subgroup, we assessed the stability of the fitted normative models using bootstrapping. As expected, model variance increased markedly when the sample size dropped below approximately 250 scans (Bozek et al., 2023). A QC threshold of 2.7 was, therefore, selected, as it ensured stable normative model estimates while retaining a sufficient sample size (n = 314). Visual inspection further confirmed the high quality of all data with QC scores above this threshold.

#### 2.4.2 Harmonization on volumetric measurements

After the categories were defined, data from each category were harmonized using the ComBat-GAM method (Pomponio et al., 2020), which extends the original ComBat algorithm (Johnson et al., 2007) by incorporating generalized additive models to flexibly adjust the nonlinear effect of age while removing site-related variance. This model was run by using gestational age as a smoothing spline; however, unlike Pomponio et al. (2020), we do not adjust for intracranial volume, as it changes dramatically over the gestational period. Harmonization was performed independently for each of the four nested quality datasets: All, Poor+, Accept+, GroundTruth.

#### 2.4.3 Normative models specification and centile trajectories

As outlined by Marquand et al. (2016), the objective of normative modeling extends beyond simply estimating a regression curve that captures the population average. It also involves determining the centiles of the distribution for each brain phenotype, allowing quantification of individual deviations from the reference model. In this study, we employed the generalized additive model for location, scale, and shape (GAMLSS) framework to analyze and characterize how each brain feature, within each QC subgroup, evolves with age as fetuses progress through gestation. This approach, originally introduced by Stasinopoulos and Rigby (2008), provides a robust and flexible framework for normative modeling. We adopted the implementation of GAMLSS developed by Dinga et al. (2021), which builds upon the R package described in Stasinopoulos and Rigby (2008). This implementation includes automated parameter tuning to optimize model fitting. In our analysis, GAMLSS models were fit using the Sinh–Arcsinh (SHASH) distribution with cubic spline smoothing, and where two parameters out of four were modeled as a function of gestational age, namely location *μ* scale *σ*. Skewness and kurtosis were estimated from the data, but were made independent from gestational age, following Bozek et al. (2023) and Dinga et al. (2021).

Within each QC subgroup, we fit a GAMLSS model to estimate the median trajectory as well as the centiles trajectories. Gestational age was rounded to two decimal places to stabilize fits and align ages across prediction curves. We assessed the stability of the GAMLSS fitting across different population samples via bootstrapping: for each QC subgroup, we fit 50 GAMLSS models from different bootstrap-resampled populations from the previously harmonized data, and reported the average and standard deviation across the centiles predicted from these models at each gestational age.

#### 2.4.4 Metrics

To quantify how much a subgroup’s predicted curve at a given centile deviates from the GroundTruth curve at the same centile for a given feature, we computed the mean *E*_1_ error as proposed in Bozek et al. (2023). The error *E*_1,_*_p_*(*a*) is defined as the distance between the curves of two GAMLSS models at centile *p* and given gestational age *a*:

$$E_{1,p}\left(a\right)={\hat{C}}_{p}^{QC_{\text{subgroup}}}\left(a\right)-{\hat{C}}_{p}^{QC_{\text{GroundTruth}}}\left(a\right),$$

where ${\hat{C}}_{p}$ represents the predicted measure at centile *p*, and $QC_{\text{subgroup}}$ denotes the specific quality tier being compared against the baseline $QC_{\text{GroundTruth}}$ . For each feature×subgroup×centile combination, we summarized a given centile’s deviation (or bias), by computing the ${\text{E}}_{1}$ mean error averaged across gestational ages, namely

$${\text{E}}_{1,p}=E_{a}\left[E_{1,p}\left(a\right)\right],$$

where *a* is sampled on a uniform grid of gestational ages between 20 and 39 weeks.

${\text{E}}_{1,p}>0$ indicates that a subgroup’s curve lies above the ground truth’s curve at the same centile (higher predicted values), while an ${\text{E}}_{1,p}<0$ indicates that a subgroup’s curve lies below that of the ground truth at the same corresponding centile (indicating lower predicted values). Values of ${\text{E}}_{1,p}\approx 0$ indicate no systematic bias. As the scale of ${\text{E}}_{1}$ varies with the size of the structure being investigated, we also reported a **normalized** ${\text{E}}_{1}$ error:

$${\text{nE}}_{1,p}=E_{a}\left[\frac{{\hat{C}}_{p}^{QC_{\text{subgroup}}}\left(a\right)-{\hat{C}}_{p}^{QC_{\text{GroundTruth}}}\left(a\right)}{{\hat{C}}_{p}^{QC_{\text{GroundTruth}}}\left(a\right)}\right].$$

#### 2.4.5 Statistical comparisons

Statistical analysis was run on the ${\text{E}}_{1}$ error distribution estimated via the 50 bootstrapped GAMLSS models. For each brain feature, centile, and QC subgroups, we tested two hypotheses. First, to assess the **presence of bias in a given subgroup**, we used a Wilcoxon rank-sum test (non-parametric and non-paired test). Namely, we tested whether the ${\text{E}}_{1}$ error between a given subgroup and the GroundTruth subgroup significantly differed from zero. Secondly, we tested whether increasing the QC stringency (e.g. from All to Accept+) would lead to statistically significant differences in ${\text{E}}_{1}$ errors between the subgroups. To do so, we assessed the **difference across QC subgroups** using a Wilcoxon signed-rank test.

### 2.5 Disentangling the impact of sample size and image quality

Up to this point, the proposed design did not allow us to separate the impact of data quality from that of sample size on a GAMLSS model, because QC involved removing data points below a given threshold, leading to nested samples of decreasing size as quality thresholding became more stringent (e.g. *n* = 314 for GroundTruth vs *n* = 635 for All).

To disentangle these effects, we needed to control for both the number of data points included in a given sample (${\text{n}}_{\text{samp}}$ ) and for the data quality of the sample. To achieve this, we implemented a weighted bootstrapping strategy. For a data point *i* with quality score *q_i_*, the resampling probability was defined as

$$p_{i}\left(\lambda \right)=\frac{\exp\left(\lambda q_{i}\right)}{\sum_{j=1}^{N}\exp\left(\lambda q_{j}\right)}$$.

Here, *λ* is a tuning parameter. The expected mean quality of the bootstrap sample was then

$$\mu \left(\lambda \right)=\sum_{i=1}^{N}\;p_{i}\left(\lambda \right)q_{i}.$$

By solving the root equation μ(λ)=q* for *λ*, where q^*^ is the desired target quality of the sample, the bootstrap resampling scheme yielded (in expectation) samples whose average quality matched the target.

Reweighted bootstrapping allowed us to obtain bootstrap samples of size ${\text{n}}_{\text{samp}}$ and controlled average quality q^*^. We then defined a grid of sample sizes ${\text{n}}_{\text{samp}}$ ∈{150,250,314} (314 is the size of the GroundTruth sample) and quality levels q*∈{2.0,2.5,3.0,3.25}. Note that setting q^*^ far above the maximum average quality implies that the bootstrapping will sample many times the same high-quality points to achieve the constraint, leading to a distorted distribution. For each of the 12 combinations, we generated 20 bootstrap samples, each of which was used to fit a GAMLSS model. This procedure was repeated for each brain feature and yielded an array of models fit on data with varying sample sizes and average quality.

We then quantified the impact of ${\text{n}}_{\text{samp}}$ and quality on the ${\text{E}}_{1}$ error distributions at the 5th, 50th, and 95th centiles across brain structures. To this end, we fit a linear mixed-effects (LME) model with the following structure:

$${\text{feature}}_{p}∼age+age^{2}+q\times {\text{n}}_{\text{samp}}+\left(1{\text{|bootstrap}}_{id}\right),$$

where residuals were assumed to be Gaussian and random intercepts were fit for each bootstrap repetition (${\text{bootstrap}}_{id}$ ). We also compared this model with an alternative specification in which the variance of the residuals was weighted according to ${\text{n}}_{\text{samp}}$ , following the hypothesis that estimation variance decreases with increasing sample size. Specifically, we assumed the residual error associated with observation i distributed as

$$\varepsilon_{i}∼N\left(0,\sigma^{2}\delta_{{\text{n}}_{\text{samp}}\left(i\right)}^{2}\right),$$

where $\delta_{{\text{n}}_{\text{samp}}\left(i\right)}^{2}$ denotes the variance multiplier associated with each ${\text{n}}_{\text{samp}}$ level. The significance of the estimated coefficients was assessed using Wald *t*-tests. We report effect sizes (*β* coefficients), *p*-values, and normalized effect sizes. Because the regression coefficients *β* are tied to the scale of the variables they model—expressing average change per unit change in the predictor—and because brain features have markedly different volumes, normalized effect sizes were computed by dividing each coefficient by the average feature volume. Multiple comparisons were corrected using Bonferroni correction across centiles.

#### 2.5.1 Code

All analyses were implemented in R using gamlss, mgcv, MASS, dplyr, tidyr, nlme, rstatix, and ggplot2; and in Python using pandas, numpy, neuroHarmonize, and matplotlib. Data tables (CSV files) and the code used to analyze and visualize the data are, respectively, available on Zenodo and GitHub.

## 3 Results

For the sake of space and clarity, we report the main results using three structures of interest that are representative of the results obtained on the other structures: cerebral white matter, lateral ventricles, and cerebellum. We report the results for the other structures as Supplementary Material.

### 3.1 Harmonization can obfuscate QC-related effects

#### 3.1.1 QC-related effects on unharmonized trajectories

**Fig. 4.**
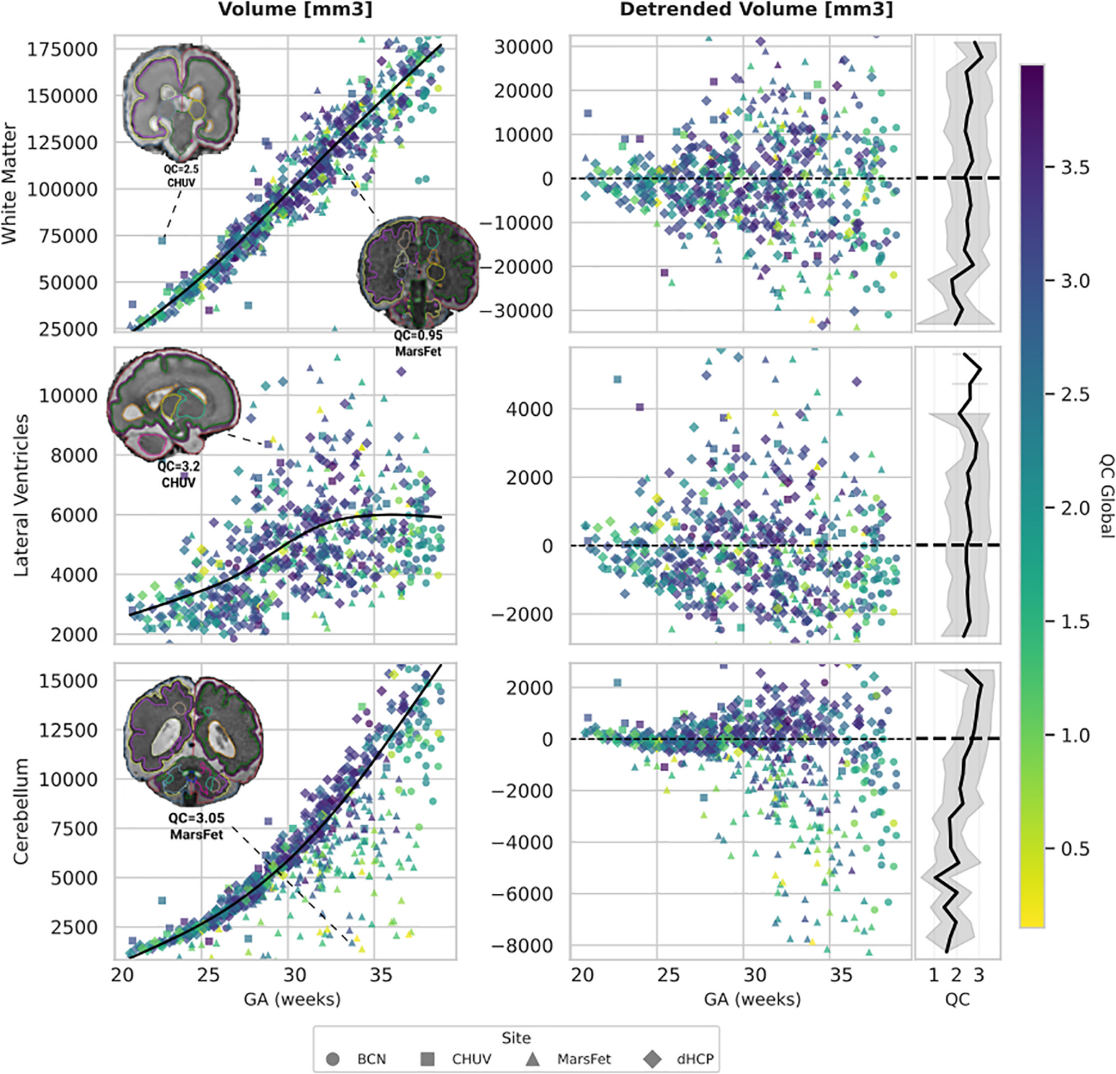
Influence of QC on raw data. (Left) Volume as a function of gestational age for raw volumes (not harmonized), for the three structures of interest. The color of the points encodes the QC level (yellow as low quality, dark blue as excellent quality). The black line is a spline fit on the GroundTruth data*.* (Middle) Detrended volume, with the spline average trend subtracted from the original volume data. (Right) Average data quality (with standard deviation) marginalized across the y-axis of the detrended volume plot.

We first look at the raw data (without harmonization) to understand whether effects related to quality can already be observed there. Figure 4 presents scatter plots of raw volume data as a function of gestational age for three representative structures, with QC scores indicated by color. The right column shows detrended plots (subtracting the average trajectory) alongside the average quality marginalized across detrended volumes.

This visualization clarifies that there is no simple relationship between QC scores and distance to the mean trajectory (residuals from regression). Poor-quality images do not systematically produce outlying data, and conversely, high-quality images can correspond to data samples located relatively far from the mean. Moreover, we observe marked variation across structures in how QC impacts the data distribution relative to gestational age. While lower QC values visibly associate with reduced cerebellar volumes ($R^{2}=0.21)$ , this pattern does not hold for lateral ventricles or white matter—particularly evident in the detrended plots (right column), where correlations between detrended volume and QC are $R^{2}<0.01$ . Results in Supplementary Section S2 (Supplementary Fig. S1) confirm these observations for the remaining structures. Overall, data points with lower QC data points *can* bias unharmonized trajectories, as confirmed by the linear association between data quality and detrended cerebellar volumes. However, this does not occur systematically across all structures.

#### 3.1.2 Impact of QC on harmonization

**Fig. 5.**
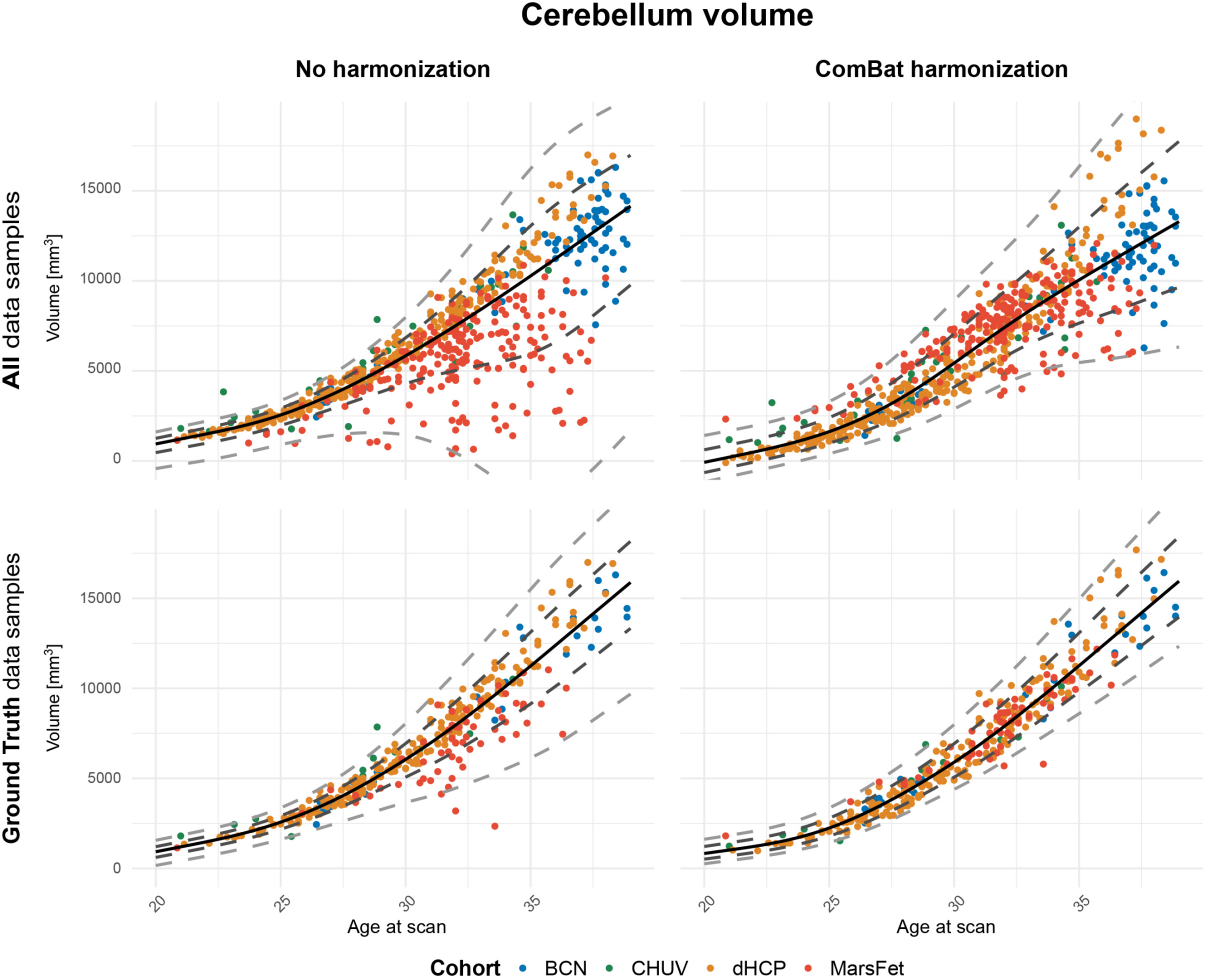
Interaction between QC and harmonization. Normative curves at centiles 1st, 5th, 50th, 95th, and 99th, for the cerebellum, fit on all data (top row), or on the GroundTruth subgroup (bottom row), and featuring raw (left) or harmonized (right) data.

We then examine the critical interaction between QC and harmonization, and its effect on normative models built using data harmonized using different threshold on QC score. Figure 5 shows normative curves fit on all data (top row) versus the GroundTruth subgroup (bottom row), without (left column) and with (right column) harmonization, for the cerebellum. As expected, harmonization clearly reduces data spread around the median trajectory and yields centile curves closer to the median. This effect is more pronounced for all data (top row) than for the GroundTruth subgroup (bottom row), since the former includes poor-quality measurements located far from the median trajectory (top left plot). Critically, when harmonization is applied in the presence of poor-quality measures (top row), the harmonization actually attempts to account for QC-related effects entangled with site effects. This leads many poor measurements to shift close to the median trajectory (top right), specifically within the 5th–95th centile curves typically used to identify abnormal data in normative models. We further investigate this effect in Supplementary Section S3 (Supplementary Fig. S2), where we quantify the effect of different QC levels on the harmonization, confirming that the harmonization absorbs some of the quality-related variability in the data. Similar patterns emerge across other structures (Supplementary Fig. S3).

These results illustrate the substantial impact of QC on normative curves and its non-trivial interaction with harmonization.

### 3.2 Poor-quality images can bias normative trajectories

**Fig. 6.**
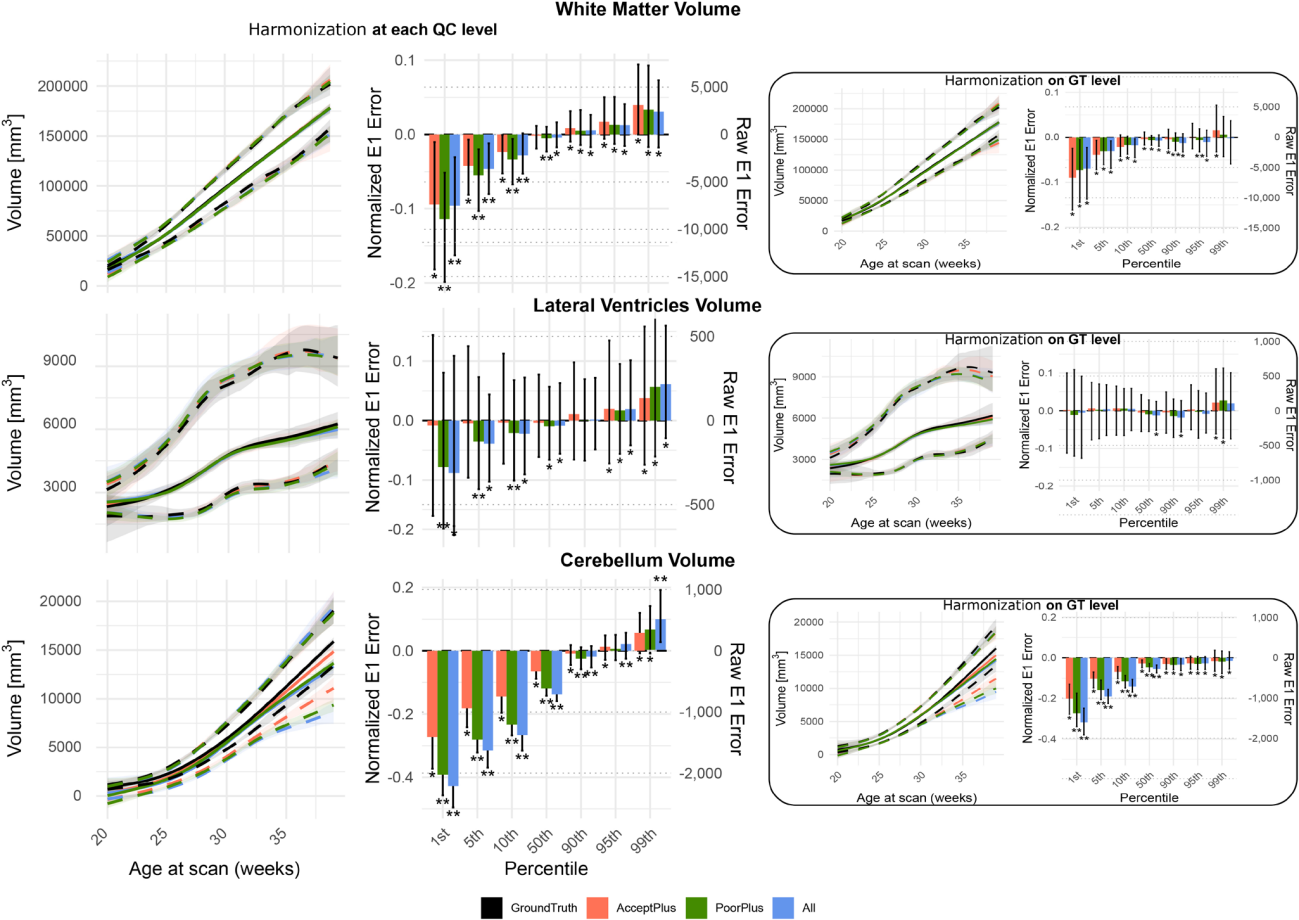
Impact of data quality on normative curves. First column: Bootstrapped curves with 95% confidence intervals for the 5th, 50th, and 95th centiles for data harmonized at each QC level. Second column: ${\text{E}}_{1}$ error distribution with 95% confidence intervals computed for 1st, 5th, 10th, 50th, 90th, 95th, and 99th centiles for data harmonized at each QC level. The Third and Fourth columns reproduce the first two columns, but are obtained using the harmonization computed on the GroundTruth data and applied to all QC tiers. A single * indicates that the mean is significantly different from 0, whereas ** indicates a significant difference from 0 and from the other quality subgroups.

Having established that harmonization can partially mask quality-related effects, we now quantify the bias that QC introduces in the normative curves themselves. Figure 6 illustrates the impact of image quality on the normative curves for white matter, lateral ventricles, and cerebellum volume. The results for the remaining structures are shown in Supplementary Section S5. The first observation from Figure 6 is the progressive impact of image quality on centile curves: deviations from the GroundTruth subgroup increase as lower-quality data are included (Accept+< Poor+< All). The second striking observation is that the impact of image quality on the normative trajectories varies across structures.

For the white matter volume, the impact of image quality on the median trajectory (50th centile) is limited, and QC biases more strongly the lower centiles (1st, 5th, and 10th) than the higher centiles (90th, 95th, and 99th). For the lateral ventricles volume, the confidence intervals around the centile curves are substantially wider, indicating greater variability across bootstrap samples compared with white matter. As for white matter, the effect on the median trajectory is small, but increases for more extreme centiles. In contrast, cerebellar volume shows an asymmetric effect of image quality relative to the median trajectory. Lower centiles exhibit a strong negative bias, whereas higher centiles are almost not affected. Notably, the effect remains negative and pronounced for the median trajectory. Overall, these results indicate that the impact of image quality is not uniformly distributed around the median: the inclusion of lower-quality data introduces a systematic bias, manifesting as a downward shift affecting both lower centiles and the median trajectory.

These conclusions still hold even when controlling for differences in the QC level of the data used for the harmonization. Instead of harmonizing the data at each QC level as was done in the left columns of Figure 6, the right panels are obtained by computing the harmonization on GroundTruth level data and applying it to all QC levels, allowing us to disentangle the effect of harmonization from model fitting. The results are highly consistent.

Observations for the remaining structures (Supplementary Fig. S5) are also consistent with these patterns. The effect of QC resembles that observed for white matter in cortical gray matter, basal ganglia, and thalamic volumes; it is similar to that of lateral ventricles for eCSF, and comparable with cerebellar volume for the brainstem. Finally, we reproduced the experiment without the dHCP dataset (Supplementary Section S7) to confirm that our observations and conclusions are not driven by this specific dataset.

### 3.3 Observed bias is independent of sample size variations

To confirm that data quality is the main factor inducing the observed bias and that it is not a consequence of an interaction with the number of samples used to fit the normative model, we carried out an experiment using weighted bootstrapping to build populations of size ${\text{n}}_{\text{samp}}\text{​}\in \left\{150,250,314\right\}$ (${\text{n}}_{\text{samp}}\text{​}=314$ being the sample size of the highest quality level) and quality q∈{2.0,2.5,3.0,3.25}. To disentangle the contributions of ${\text{n}}_{\text{samp}}$ and *q*, we generated 20 bootstrap samples for each brain structure, and $\left({\text{n}}_{\text{samp}},q\right)$ combination, which were used to fit different normative models. We then used an LME model to quantify the relative effect of ${\text{n}}_{\text{samp}}$ and *q*.

**Fig. 7.**
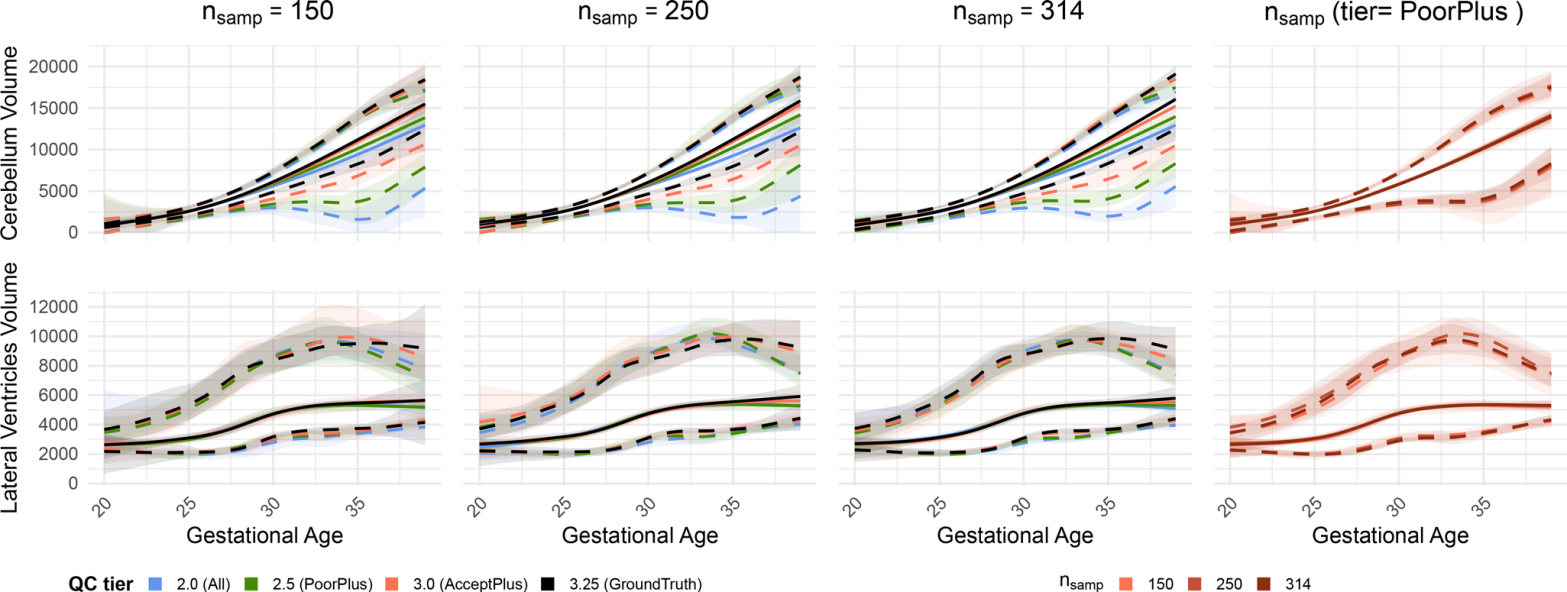
Bootstrapping samples confirm a consistent bias induced by QC. Weighted bootstrapping allows obtaining samples with a controlled ${\text{n}}_{\text{samp}}$ as well as *q*. The first three columns show normative centile curves at various ${\text{n}}_{\text{samp}}$ (150,250,314 ) for different *q* (2.0,2.5,3.0,3.25 ), and the last column shows all ${\text{n}}_{\text{samp}}$ for a fixed quality (Poor+ q=2.5 ). Dashed lines represent outer centiles (5th and 95th), respectively, and full lines the 50th centile.

Figure 7 clearly shows that the bias affecting the centile curves is consistent across ${\text{n}}_{\text{samp}}$ . This is confirmed in the right-most column of the figure, where we superimpose the curves obtained with different ${\text{n}}_{\text{samp}}$ for a given QC subgroup *q*. The observations for the other brain structures in Supplementary Section S6 (Supplementary Fig. S6) are consistent.

Quantitative results from the LME analysis are presented in Table 2 (with its extended version given in Supplementary Table S2). The results indicate that there is a consistently significant effect of QC on the estimated centiles. The effect size is orders of magnitude larger than the effect size of ${\text{n}}_{\text{samp}}$ . Note that this difference is (partially) offset by the difference in scale between the variables.

**Table 2.** Effect size from an LME model shows that the impact of QC on estimated centiles is consistent, large, and asymmetrical.

		QC effect size		${\text{n}}_{\text{samp}}$ effect size		Interaction
Region	Centile	Raw	Relative	Raw	Relative	Eff. size
eCSF	5th	5026.79	0.094	−11.88	$-2.21\times 10^{-4}$	4.41
	50th	3690.43	0.051	−6.54	$-9.07\times 10^{-5}$	2.84
	95th	3773.77	0.040	—	—	—
Cortical gray matter	5th	1184.26	0.031	−11.15	$-2.97\times 10^{-4}$	2.76
	50th	1418.15	0.030	—	—	—
	95th	1070.19	0.018	4.90	$8.45\times 10^{-5}$	—
White matter	5th	6754.08	0.087	33.39	$4.32\times 10^{-4}$	−13.87
	50th	1790.87	0.019	15.40	$1.65\times 10^{-4}$	−5.74
	95th	728.82	0.007	—	—	—
Lateral ventricles	5th	170.82	0.060	—	—	—
	50th	52.00	0.012	—	—	—
	95th	112.79	0.015	—	—	—
Cerebellum	5th	2470.67	0.680	3.04	$8.33\times 10^{-4}$	−1.00
	50th	607.11	0.097	−0.70	$-1.12\times 10^{-4}$	0.30
	95th	148.31	0.019	—	—	0.34
Basal ganglia	5th	114.32	0.031	—	—	—
	50th	−38.92	−0.008	−0.29	$-6.19\times 10^{-5}$	0.11
	95th	−487.11	−0.082	—	—	—
Brainstem	5th	665.91	0.238	—	—	—
	50th	163.37	0.044	0.15	$4.09\times 10^{-5}$	—
	95th	45.80	0.011	0.55	$1.27\times 10^{-4}$	—
Thalamus	5th	22.62	0.007	−0.82	$-2.67\times 10^{-4}$	0.24
	50th	−17.55	−0.005	—	—	—
	95th	−94.26	−0.021	1.24	$2.78\times 10^{-4}$	−0.32

Dashed cells represent non-statistically significant results (p≥0.05), raw effect sizes are the slopes (*β*) of the fit LME model.

To contextualize these effect sizes, consider that in our experimental design, quality levels differ incrementally by 0.5 units (e.g., from 2.0 to 2.5), while sample sizes differ by 100 scans (e.g., from 150 to 250). For eCSF’s 50th centile, a 0.5-unit increase in average QC corresponds to a 2.6% volume increase (0.051×0.5 ), whereas adding 100 scans yields a 0.9% volume decrease ($-9.07\times 10^{-5}\times 100$ ). The interaction term also implies that the impact of QC will be even stronger in larger sample sizes. This illustrates how QC has a substantially larger impact than sample size on the observed biases. Crucially, these systematic shifts in the underlying centile curves directly alter the clinical utility of the normative model, since even a slight change in the centile curve could change the classification of individual patients as *normal* or *abnormal*.

In summary, our findings firmly establish that data quality introduces a systematic bias into normative centile curves: irrespective of the amount of data used to fit a model, the average data quality has a systematic and consistent biasing effect. Furthermore, except for the basal ganglia and thalamus, this effect shows an asymmetric and non-uniform downward bias induced by lower-quality data. Specifically, those lower-quality data generally draw the estimated volumes down, and have a stronger effect on inferior centiles than on upper ones.

## 4 Discussion

### 4.1 Quality-induced bias in the context of early brain development

Previous studies focusing on QC have demonstrated that poor data quality (often driven by motion) biases image-derived biomarkers (Backhausen et al., 2016; Gilmore et al., 2021; Reuter et al., 2015). However, these analyses are typically static, comparing adult groups without explicitly accounting for developmental trajectories. In fetal imaging, such an approach is not relevant, as rapid brain development necessitates explicit modeling of gestational age. By incorporating a temporal dimension through normative modeling, our results extend prior findings to the fetal domain and demonstrate that data quality biases not only individual biomarkers but also the estimated developmental centiles themselves. For most structures, including cortical gray matter, white matter, cerebellum, brainstem, lateral ventricles, and extra-cerebral CSF, lower-quality data tend to produce systematically lower volume estimates, with particularly strong effects observed in the cerebellum and brainstem. The direction of this effect is reversed for the basal ganglia and thalamus, where lower-quality data impact more the higher centiles than the lower ones. However, because our quality rating aggregates several quality dimensions such as tissue contrast and reconstruction artifacts into a single global score, disentangling their individual contributions to these patterns is beyond the scope of the present work.

### 4.2 Data quality can bias normative models

Our results demonstrate the critical influence of data quality on normative modeling. Including lower-quality data systematically biases estimated centiles, with the largest effects observed at the distribution extremes, particularly in the lower centiles (1st–10th). Importantly, these effects do not arise from a uniform increase in variance: poor-quality data can bias normative curves asymmetrically across centiles, and may even affect the median trajectory. Failing to exclude poor-quality data prior to fitting normative models, therefore, leads to systematic, non-trivial bias patterns in the resulting centile curves.

Although prior fetal normative studies generally report having performed QC, the absence of clearly defined and reproducible QC protocols makes it difficult to evaluate the extent to which their reported centiles may be biased. Importantly, by leveraging a continuous QC scale, we show that bias increases progressively as QC criteria are relaxed, rather than emerging only from severely degraded or obviously corrupted scans. This continuum challenges binary inclusion–exclusion approaches to QC and highlights the need for more nuanced strategies, as even moderate reductions in QC stringency can lead to systematic shifts in centile estimates.

Taken together, these results underscore the necessity of adopting standardized and transparent QC protocols at the community level. Such efforts will be essential not only to improve reproducibility and consistency across normative modeling studies, but also to support the safe translation of these models into clinical practice.

### 4.3 Clinical implications

Our findings are particularly important from a clinical perspective, as centiles are often the most actionable outputs of normative models, informing the detection of atypical developmental trajectories and clinical risk stratification (Marquand et al., 2016). This susceptibility raises concerns regarding the clinical reliability of normative models derived from datasets with insufficiently well-defined QC procedures. Our results imply that the centiles of a normative model built and deployed without accounting for data quality can be biased, potentially resulting in misclassifying pathological subjects as healthy or the reverse. For example, if we consider the 5th centile as a standard diagnostic threshold for normality, the practical consequences of this bias are substantial. For the thalamus, a subject placed at the 5th centile by the GroundTruth model would be placed above the 10th centile by the All model. For the cerebellum, the discrepancy is far larger: a subject placed at the 1st centile by the GroundTruth model would be placed near the 26th centile by the All model, a severe misclassification. Such a pronounced inflation would effectively obscure severe structural abnormalities, illustrating how a lack of stringent QC can directly compromise patient stratification and clinical decision making.

### 4.4 Bias–variance tradeoff and implications for fetal normative modeling

Our results highlight a fundamental bias–variance tradeoff in normative modeling. Recent work on normative modeling has emphasized the importance of large sample sizes for estimating reliable developmental trajectories (Bozek et al., 2023). Our results add an important nuance to this message: while sample size matters, data quality is a critical determinant of bias in normative centiles, which cannot be compensated by increasing the sample size. Using a larger number of lower-quality scans leads to centile estimates with lower variance but increased bias, whereas restricting analyses to higher-quality data reduces bias at the cost of increased variance due to smaller effective sample sizes. This tradeoff is particularly important in fetal neuroimaging, where sample sizes are typically small (n = 127 in Kyriakopoulou et al. (2017); n = 122 in Machado-Rivas et al. (2022); n = 340 in X. Xu et al. (2024)). Our bootstrapping experiments highlighted a substantial increase in variance when fewer than n = 250 samples were used to fit a normative model. However, increasing sample size at the expense of data quality could paradoxically degrade the reliability of derived growth trajectories and compromise their clinical utility as lower-quality data will bias the centiles of the normative models.

### 4.5 Interactions between image quality and segmentation accuracy

An important observation from our analyses is that, although image quality clearly biases normative centiles, disentangling its effects from those related to segmentation accuracy remains challenging. Many scans rated as lower quality do not exhibit overt segmentation failures (Figs. 1 and 4), yet their inclusion still leads to systematic shifts in centile estimates. Conversely, some scans with apparently good image quality yield poor segmentations for specific structures, as illustrated by segmentation failures for the cerebellum in a subset of the MarsFet dataset.

While it is common, even in adult neuroimaging studies, to rely exclusively on image-level QC (Backhausen et al., 2016; Esteban et al., 2017; Reuter et al., 2015; Rosen et al., 2018), the most effective QC strategy should integrate both image-based and segmentation-based quality criteria (Klapwijk et al., 2019; Monereo-Sánchez et al., 2021). Implementing such approaches is particularly challenging in the fetal imaging context; however, as there is currently no widely adopted segmentation framework, like FreeSurfer (Fischl, 2012), that could serve as a common reference standard. In the absence of such consensus, we encourage researchers developing new segmentation methods to report failure modes in a systematic and detailed manner. Such transparency would be instrumental in enabling end users to design effective QC protocols, and in accounting for the complex interplay between image and segmentation quality in downstream analyses.

### 4.6 Limitations and future work

This work currently has some limitations. First, as discussed above, our QC protocol relied exclusively on image-level assessment. Designing and releasing a standardized protocol for segmentation-level QC, pursuing the efforts initiated by Bach Cuadra et al. (2025), is an important direction for future work.

Second, while we relied on BOUNTI (Uus, Kyriakopoulou, Makropoulos, et al., 2023) for the segmentation in this work, this algorithm was only designed to handle healthy subjects. There is then a great need to develop tools able to segment both healthy and pathological subjects, as the usefulness of normative models will be in their ability to correctly flag pathological subjects as outliers, which supposes first a correct segmentation of such subjects.

Third, although our study includes the largest multi-centric fetal brain cohort to date (n = 635), this sample size remains below the recommendations for normative modeling (Bozek et al., 2023). As such, the precision of the estimation of the outer centiles might still be limited by the sample size.

Fourth, our analyses focused primarily on typically developing fetuses. While this was necessary to establish normative trajectories, the impact of quality-induced bias on pathological cases, where accurate centile placement is most critical, remains to be fully characterized.

Fifth, although we relied on state-of-the-art methods for the processing of our data (Sanchez et al., 2026), the generalizability of our findings to imaging protocols beyond fast spin echo and to other segmentation pipelines (Zalevskyi et al., 2024) should be investigated in future studies.

Finally, although our study provides evidence of a systematic downward shift of centiles due to data quality, it would be useful to confirm these findings in a more controlled setting. Introducing artificial degradations on high-quality images could provide such a framework (Loizillon et al., 2023), and would enable a finer understanding of how different dimensions of image quality (relative tissue contrast, bias field, reconstruction artifacts, etc.) might drive the biases that we described through a global image quality lens in this study. This would be a valuable direction to confirm and deepen the findings of the present work.

## 5 Conclusion

In conclusion, our results demonstrate that data quality is a major and often underappreciated source of bias in fetal brain normative modeling. While increasing sample size is commonly viewed as the primary avenue to improve the reliability of normative models, our results show that doing so at the expense of data quality can systematically distort centile estimates, particularly at the clinically relevant extremes. Quality-induced bias is subtle, structure dependent, and progressive, challenging the prevailing view of QC as a simple inclusion–exclusion procedure. Instead, shifting toward a continuous, quantitative characterization of data quality would provide a more nuanced perspective, and enable the discussion of community-accepted levels of QC stringency. Such transparent and standardized QC practices will be essential to ensure the robustness, comparability, and ultimately the clinical utility of fetal brain growth models.

## Supplementary Material

Supplementary Material

## Data Availability

Tabular data (estimated volumes data and corresponding data quality for each scan) are available on a Zenodo repository at https://doi.org/10.5281/zenodo.21485414. The code is publicly available on GitHub at https://github.com/fetpype/normative_modeling_data_quality. Sharing of the reconstructed images and segmentations requires data transfer agreements with each of the centers.

## Footnotes

1 https://www.developingconnectome.org (fourth release).

2
https://github.com/fetpype/fetpype
