# Supplementary material for: Data quality biases normative models derived from fetal brain MRI
Source: Imaging Neurosci (Camb). 2026 Aug 14;4:IMAG.a.1337. doi: 10.1162/IMAG.a.1337 (PMC13479347; doi:10.1162/IMAG.a.1337)
Supplement: Supplementary file 1 [file IMAG.a.1337_supp.pdf]

Supplementary Material

Table of contents:

S1	(p. 2)	Inclusion and exclusion criteria
S2	(p. 3)	Detrended plots on additional structures
S3	(p. 4)	ComBat-GAM harmonization
S4	(p. 8)	Iterative scan removal
S5	(p. 9)	Normative curves and E1 errors on additional structures
S6	(p. 10)	Disentangling the effect of QC and sample size on additional structures
S7	(p. 12)	Leave-dHCP-out experiment

S1 Inclusion and exclusion criteria

To establish a healthy fetal cohort, a multidisciplinary team—including specialists in neuroradiology, obstetrics, and pediatric neurology—developed exclusion criteria to ensure clinical and anatomical normality and standardization across sites. The resulting inclusion and exclusion criteria are detailed in Table S1 below.

**Inclusion criteria**
○ Sufficient MRI data quality to be processed by the pipeline (at least 3 orthogonal scans) without returning an error).
**Exclusion criteria**
○ No consent of parents (or legal representatives) to participate in the study
○ Multiple pregnancy
○ *Brain malformation:* microcephaly (-3SD), macrocephaly (+2SD), commissural anomalies (corpus callosum anomalies, septal agenesis), posterior fossa anomalies (Dandy–Walker spectrum, compressive arachnoid cysts, mega grande cisterna $>$ 12 mm, cerebellar hypoplasia, pontocerebellar malformations), clastic lesions, cortical developmental anomalies
○ Enlargement of ventricles superior than 10 mm at ultrasound
○ Extracerebral malformation (particularly cardiac or syndromic)
○ Genetic syndrome and/or chromosomal abnormality and/or deleterious mutation
○ Fetal alcoholism
○ *Detrimental perinatal event:* difficult traumatic delivery, anoxo-ischemia, respiratory distress, convulsion, maladjustment to extrauterine life
Fetal MRI sessions in the following situations were not excluded:
○ Maternal infection by cytomegalovirus, for which the infection of the fetus was ruled out by test on amniotic fluid or urine in the neonatal period
○ Presence of small (inferior to 3 mm) periventricular cysts

Table S1: Inclusion and exclusion criteria for the study subjects.

S2 Detrended plots on additional structures

Figure S1 complements Figure 4 and shows globally similar trends. When detrended, there is no obvious connection between outliers and QC.

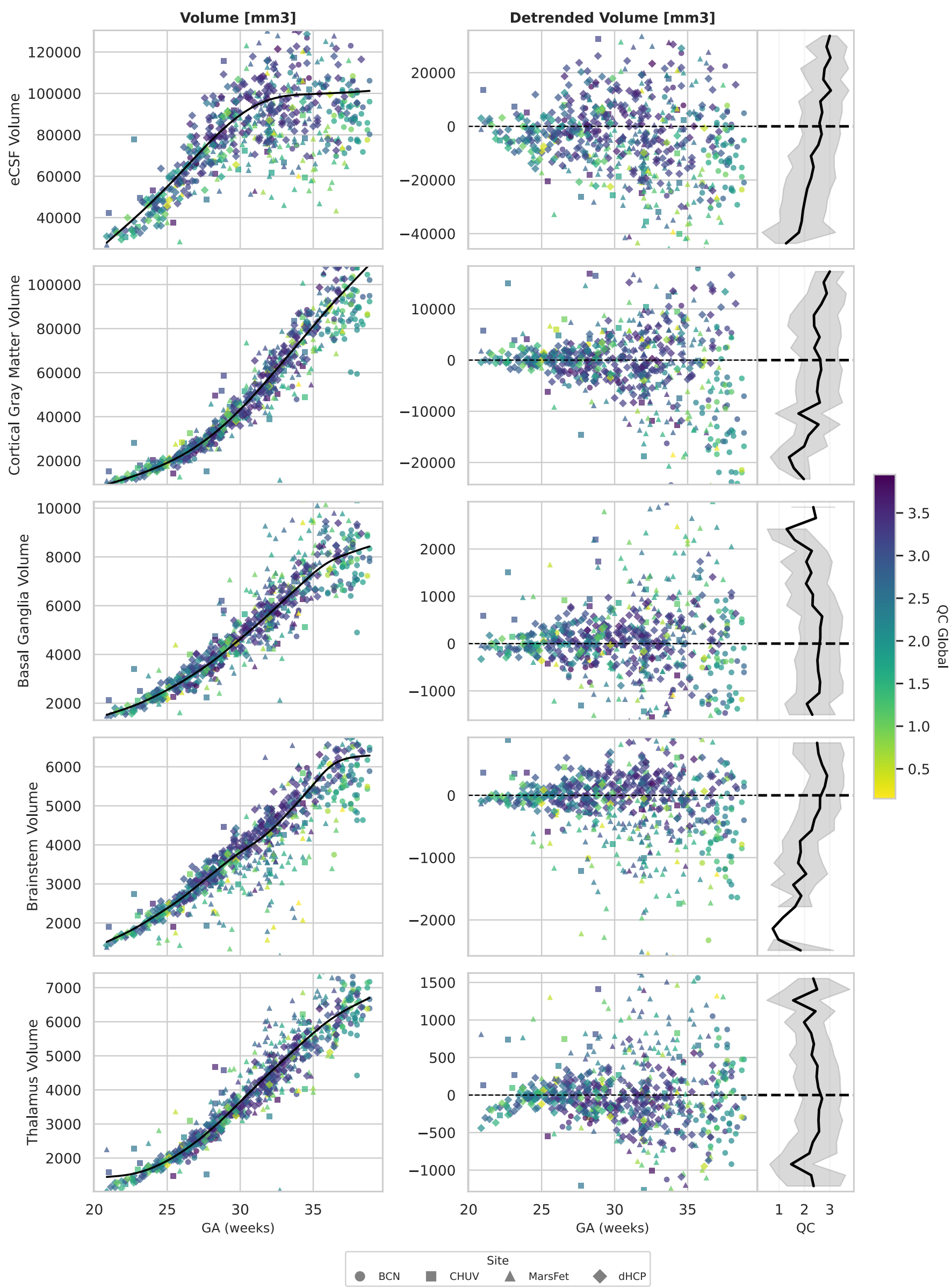

Figure S1: **Influence of QC on raw data.** *(Left)* Volume as a function of gestational age for raw volumes (not harmonized). The black line is a spline fit on the data. *(Middle)* Detrended volume, with the spline average trend subtracted from the original volume data. *(Right)* Average data quality (with standard deviation) marginalized across the y-axis of the detrended volume plot.

S3 ComBat-GAM harmonization

Pomponio et al. (2020) proposed ComBat-GAM, which we used for the harmonization of our data. We summarize here their method and resulting harmonization.

Given a volume Y_{ijk}, for a given site i, scan j and ROI k, the aim of ComBat-GAM is to remove site-related inconsistencies while retaining biological differences. The method first uses a generalized additive model (GAM) to remove the effect of age from the volume, before running ComBat (Johnson et al., 2007) on the detrended data, before finally removing the site effects on Y_{ijk}. We provide the mathematical framework closest to the computational implementation of ComBat-GAM in `neuroHarmonize` (https://github.com/rpomponio/neuroHarmonize/).

First, a non-linear GAM model is fitted to each individual ROI:

$$Y_{ijk} = f_k^*(a_{ij}) + n_{ij} \tag{1}$$

where $f_k^*(a_{ij})$ is a cubic spline fitting the volume as a function of the gestational age a_{ij} on all available data from the ROI k, and n_{ij} the residual from this fit. Note that, contrary to Pomponio et al. (2020), we do not adjust for intracranial volume, as it changes dramatically over the gestational period and would lead to unstable GAM fitting in our setting.

The fitted GAM model is then used to detrend and standardize the data:

$$Z_{ijk} = \frac{Y_{ijk} - f_k^*(a_{ij})}{\sigma_k} \tag{2}$$

where σ_k is the estimated standard deviation of the data for a given ROI k, defined as $\sigma_k = \sqrt{\text{var}\left(Y_{ijk} - f_k^*(a_{ij})\right)}$. The resulting Z_{ijk} then represent GA-detrended and standardized data.

Note that ComBat-GAM assumes that the variance σ_k does not depend on the age. While this assumption generally holds for adult data, this is not true for fetal data. We acknowledge this as a structural limitation of applying standard ComBat-GAM to fetal datasets; however, developing a novel harmonization framework featuring an age-adaptive variance parameter ($\sigma_k(a_{ij})$) is beyond the scope of the present work.

Given the standardized data Z_{ijk}, ComBat is then used to estimate site effects. This is achieved through an Empirical Bayes framework, which estimates site-wise location and scale parameters. These parameters are optimized by minimizing the posterior expected squared error of the site effect estimates, regularized by the prior. Specifically, the site-specific location (γ_{ik}^*) and scale (δ_{ik}^*) parameters are assumed to be

drawn from the following prior distributions:

$$\gamma^*_{ik} \sim \mathcal{N}(\gamma_i, \tau_i^2), \quad \delta^*_{ik} \sim \mathsf{InverseGamma}(\lambda_i, \theta_i).$$

These prior distributions were selected for their mathematical conjugacy with the Normal likelihood of the standardized data Z_{ijk}, which yields closed-form posterior estimates for γ^*_{ik} and δ^*_{ik}, without requiring numerical optimization.

Given the estimated parameters γ^*_{ik} and δ^*_{ik} for each site and ROI, the harmonized data then reads

$$Y^*_{ijk} = \frac{(Z_{ijk} - \gamma^*_{ik})\sigma_k}{\delta^*_{ik}} + f^*_k(a_{ij}) \tag{3}$$

$$= \overbrace{f^*_k(a_{ij})}^{\text{Average trend}} - \underbrace{\frac{\sigma_k}{\delta^*_{ik}}\gamma^*_{ik}}_{\substack{\text{Additive}\\ \text{shift term}}} + \overbrace{\frac{1}{\delta^*_{ik}}}^{\substack{\text{multiplicative}\\ \text{scaling term}}} \cdot \underbrace{(Y_{ijk} - f^*_k(a_{ij}))}_{\text{Detrended data}} \tag{4}$$

Here, the *average trend* does not depend on the *site*, but will differ for each QC level. The *additive shift* and *multiplicative* shift terms provide site-specific parameters, and are the main factors of the harmonization. Note that these terms are independent on gestational age.

Figure S2 below shows this decomposition computed on several QC levels. Importantly, the dimensions are not independent, as the shift and scales are applied on the detrended data, and we see on the left column that the average trend changes with QC levels. On this Figure, we observe that QC affects not only the average trend, but also the strength of the required harmonization. In almost all cases, the magnitude of the scale parameter decreases across sites as QC increases. In structures like the cerebellum and the brainstem, the shift also decreases with higher data quality, whereas no consistent trend is observed across sites for the remaining structures. The fact that the relative strength of harmonization changes with QC levels confirms that harmonization can absorb some variance in the data that is due to data quality.

Looking further at the impact of these different levels of QC on the derived normative models, Figure S3 complements the results of Figure 5 and shows how QC and harmonization interact in non-trivial ways. Although the observed effect is generally less dramatic than what was highlighted for the cerebellum, we still observe large differences in predicted centiles, especially for the brainstem, where centiles remain spread independently of harmonization when data of poor QC levels are included in the fit.

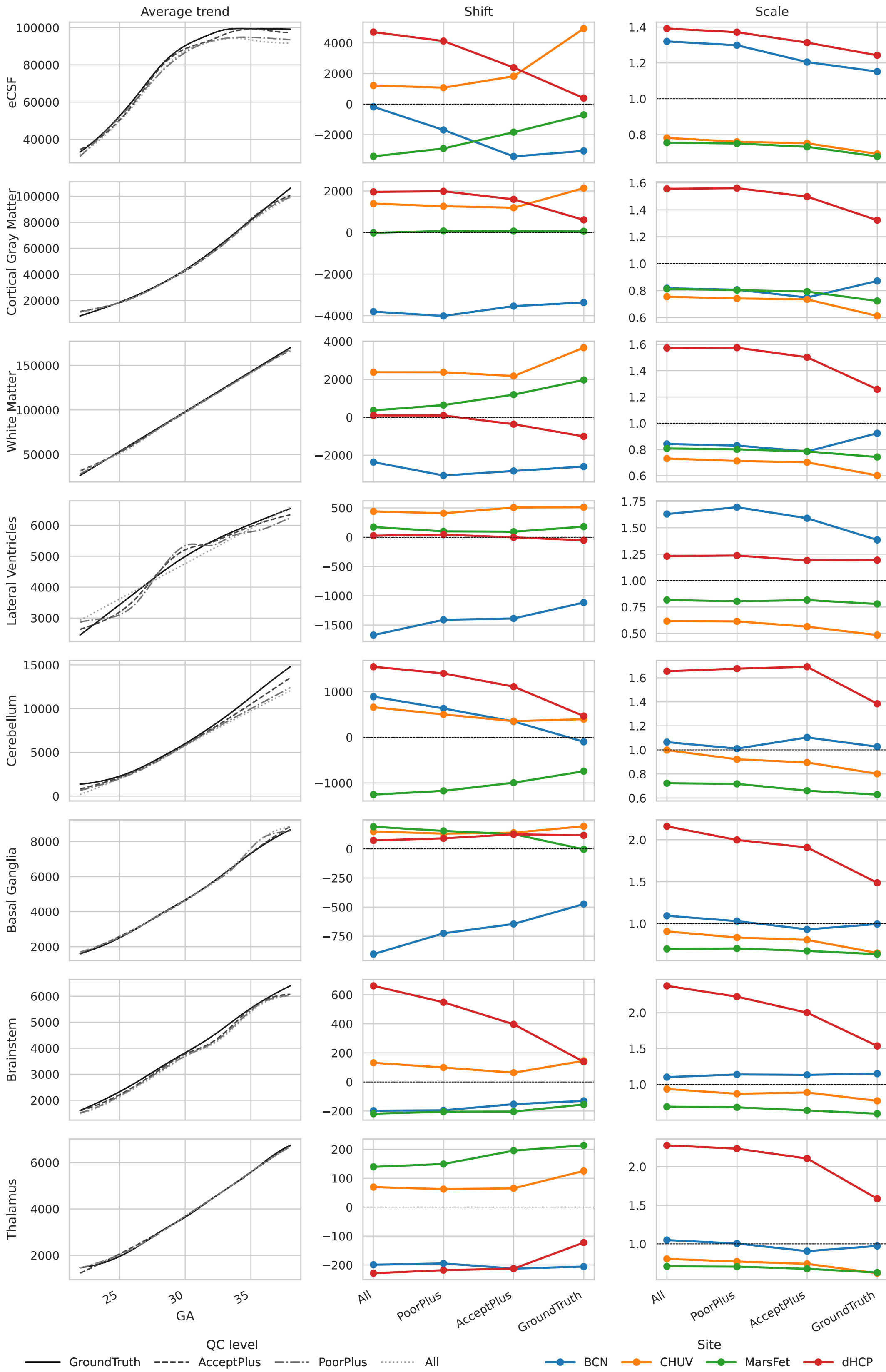

Figure S2: Average trend, additive shift and multiplicative scale parameters across all structures of interest and quality levels, obtained from the decomposition of Equation (4).

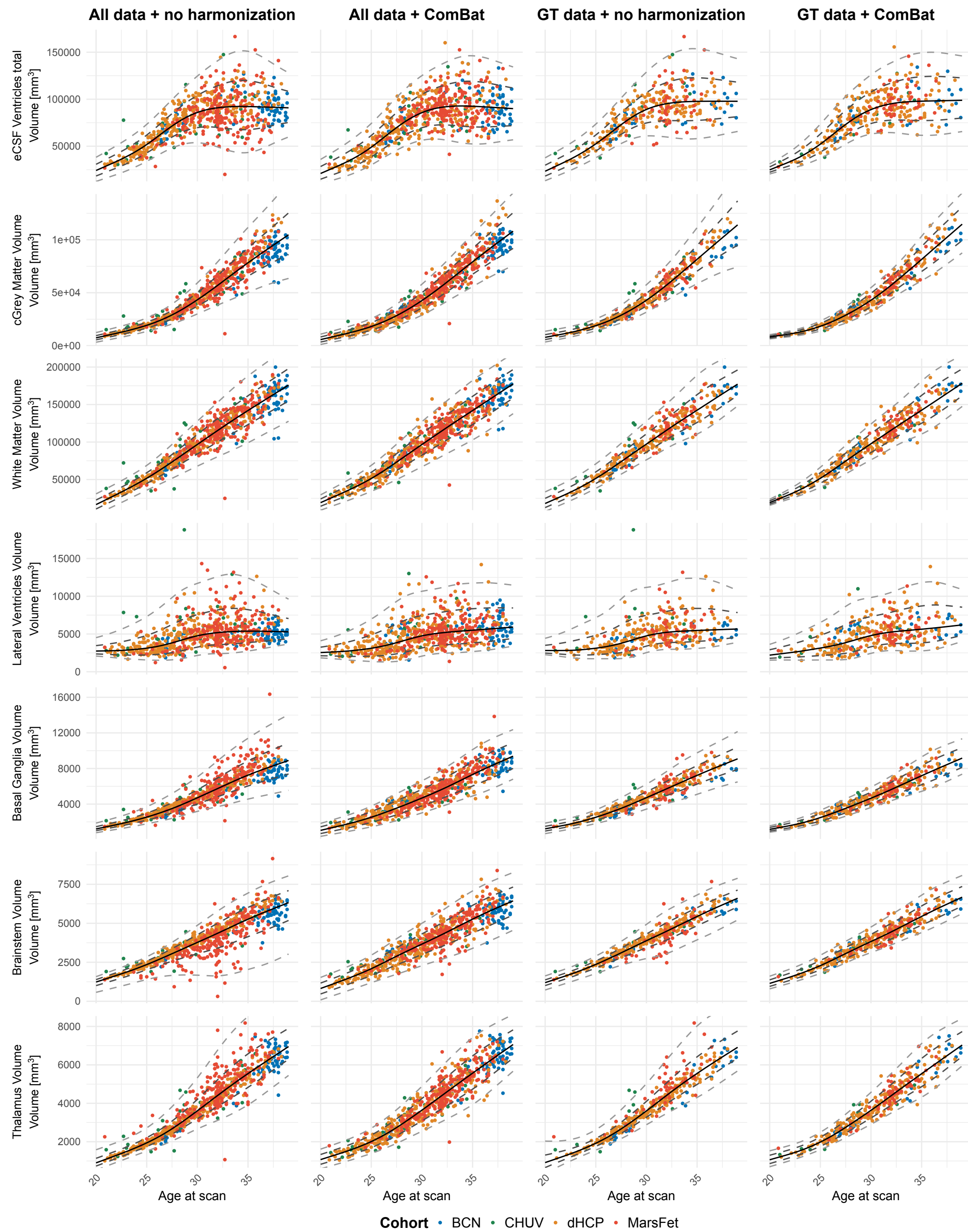

Figure S3: Normative curves at various centiles (1st, 5th, 50th, 95th and 99th) for different structures of interest, fit using either all data or QC-ed data (bottom row), and featuring raw or harmonized data.

S4 Iterative scan Removal

For this experiment, reported in Figure S4 we started from the entire pool of 635 scans. We then sorted the data according to QC and iteratively removed the data of lowest quality, 5 by 5 – quality is illustrated on the bottom right of Figure S4. The data were then harmonized on the remaining samples, and then 10 bootstrap samples were used to estimate the variance due to population sampling on the fit GAMLSS models. The normalized E1 error was then computed between the curve obtained at n_{samp} and the original curve fit using all scans.

The results, reported for the 5th and the 50th centile provide a more granular version of Figure 6 (and of Figure S5 in the next section), showing that a difference appears when samples of lower quality are removed. The results also highlight an instability in the model fits when going to $n_{\text{samp}} < 250$, with a drastic increase in variance across models. Setting the ground truth criterion at $QC = 2.7$ allows us to remain far from this high variance zone.

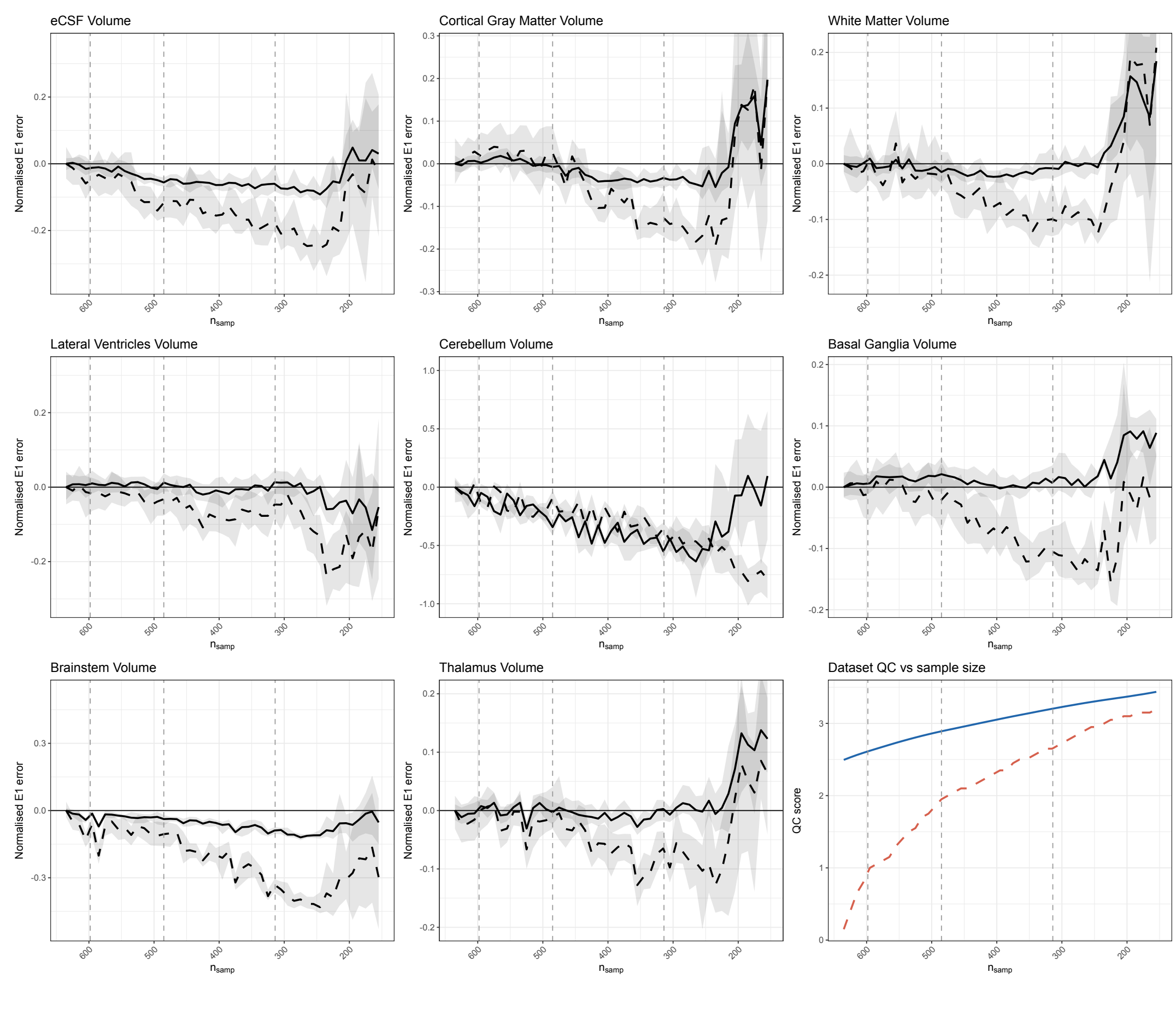

Figure S4: Normalized E1 error against $n_{\text{samp}} < 250$ when iteratively removing scans of low QC values. Thresholds denote the different levels of inclusion criteria discussed in the paper. The vertical dashed lines correspond to the `Poor+`, `Accept+` and `GroundTruth` thresholds.

S5 Normative curves and E1 errors on additional structures

Figures S5 complements Figure 6 and confirms the strong effect of QC on biasing normative centiles across all brain structures considered.

The observations for the structures here generally follow three kinds of trends: the impact of QC is similar to white matter for cortical gray matter, basal ganglia and thalamus volumes — we observe a small but systematic spread; it is similar to lateral ventricles for eCSF — where we see a very large variance across bootstraps, especially in the E1 error —, and similar to cerebellum for the volume of the brainstem — where a strong bias is observed in lower centiles.

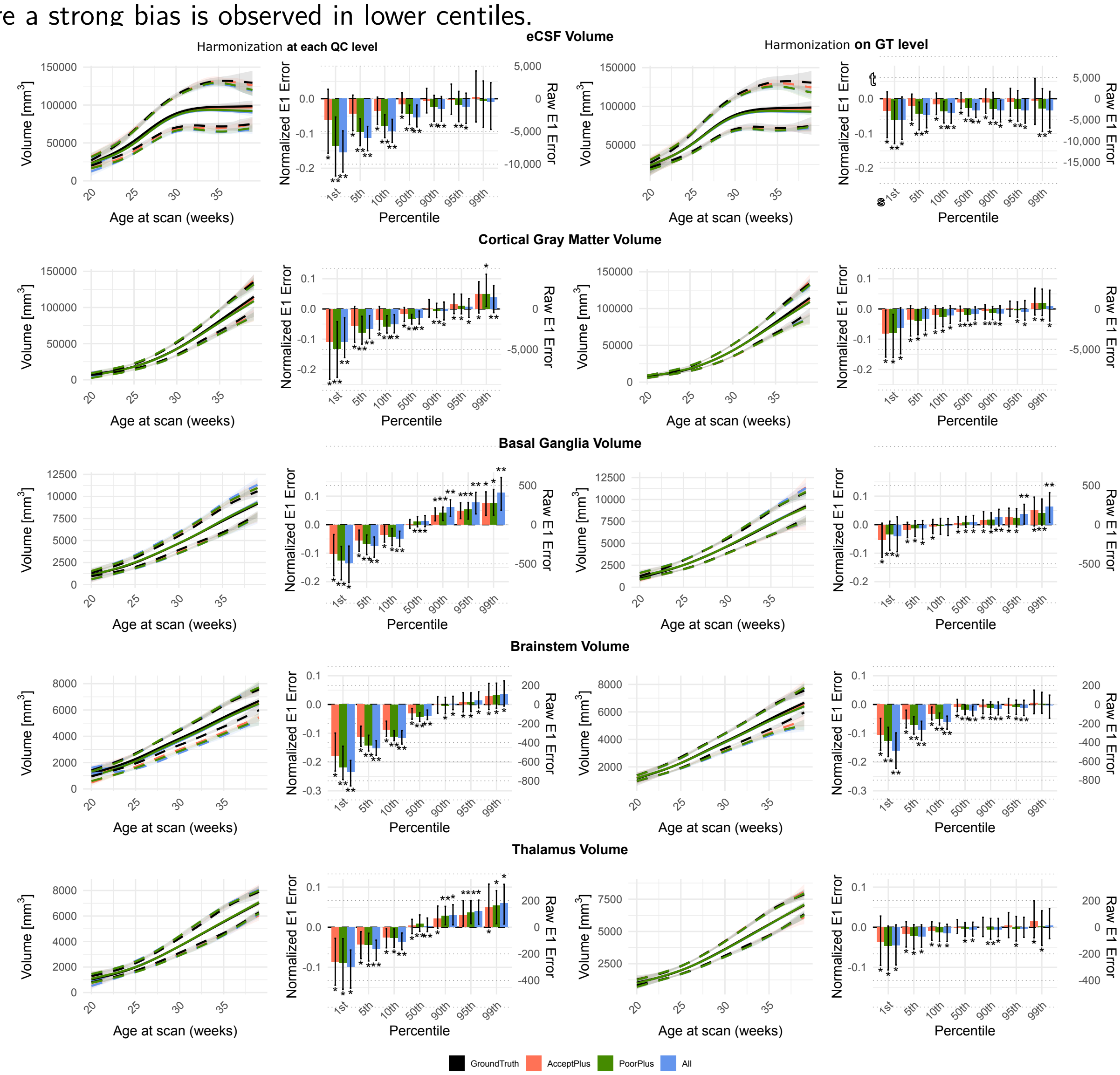

Figure S5: **Impact of data quality on normative curves for the rest of brain ROI.** This complements Figure 6, showing the bootstrapped curves for the 5th, 50th and 95th centiles as well as E1 errors, for two settings: harmonization at each QC level (left) and harmonization on GT data (right).
A single ⋆ indicates that the mean is significantly different from 0, whereas ⋆⋆ indicates a significant difference from 0 and from the other quality subgroups.

S6 Disentangling the effect of QC and sample size on additional structures

Figure S6 extends the ablation study in Figure 7 and further confirms the results observed. Across all brain structures and sample sizes, there is a bias due to the average QC of the data used to fit the normative model. Table S2 extends Table 2, also showing p-values, as well as non-significant results.

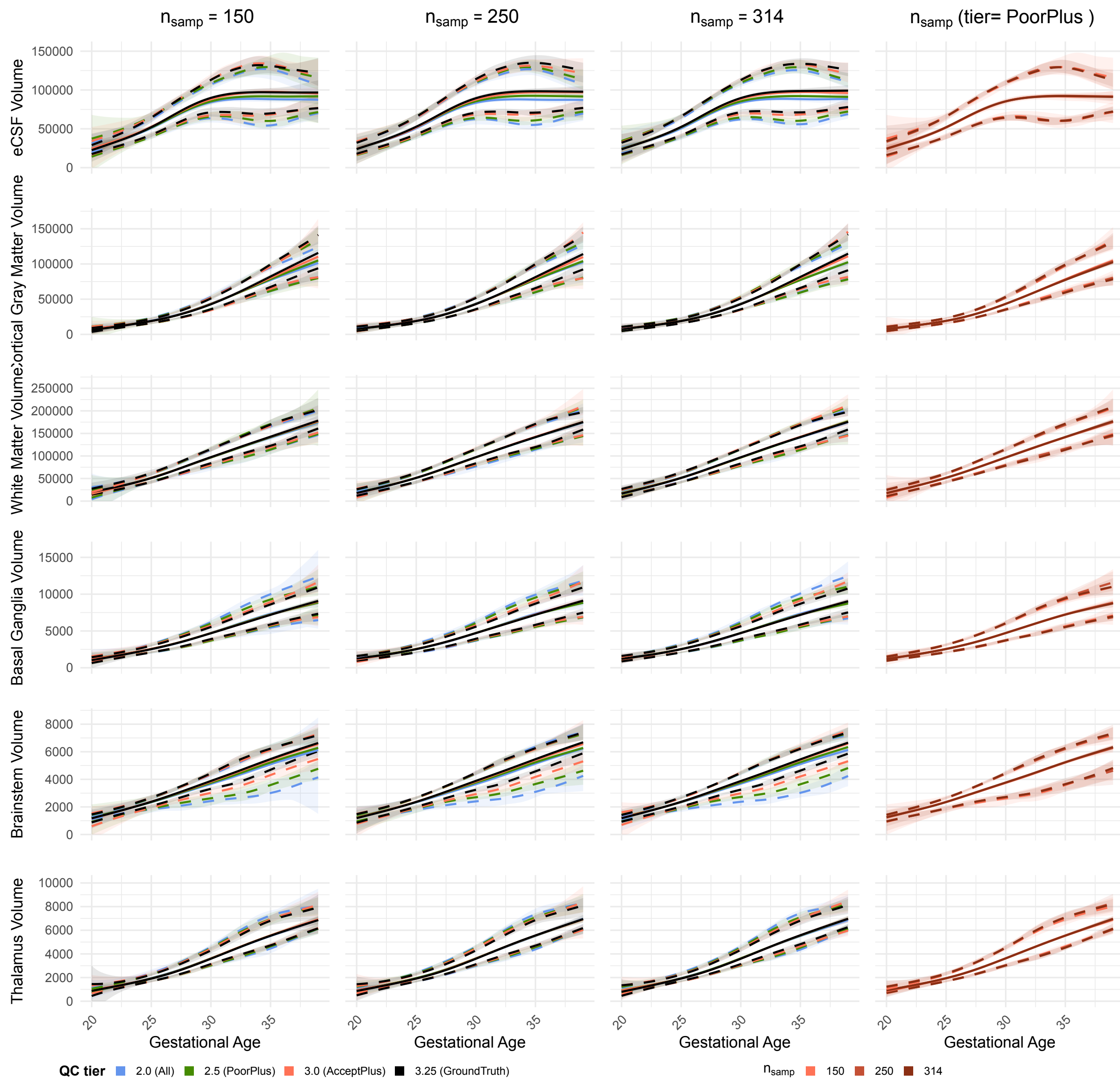

Figure S6: Bootstrapping samples with a given n_{samp} and average quality q for additional structures. Extended version of Figure 7.

**Region**	**Centile**	**QC**		$\mathbf{n_{samp}}$		**Interaction**		**Rel. eff. size**		$\mathbf{n_{samp}}$	**variance**	
		Eff. size	p-val.	Eff. size	p-val.	Eff. size	p-val.	QC	n_{samp}	150	250	314
eCSF	5th	5026.79	$< 10^{-6}$	-11.88	2.50×10^{-4}	4.41	2.04×10^{-4}	0.094	-2.21×10^{-4}	1.00	0.83	0.79
	50th	3690.43	$< 10^{-6}$	-6.54	1.16×10^{-2}	2.84	1.88×10^{-3}	0.051	-9.07×10^{-5}	1.00	0.89	0.88
	95th	3773.77	$< 10^{-6}$	-3.02	1	2.04	6.00×10^{-1}	0.040	-3.20×10^{-5}	1.00	0.88	0.79
Cortical Gray Matter	5th	1184.26	$< 10^{-6}$	-11.15	$< 10^{-6}$	2.76	6.12×10^{-5}	0.031	-2.97×10^{-4}	1.00	0.82	0.74
	50th	1418.15	$< 10^{-6}$	-0.89	1	0.01	1	0.030	-1.89×10^{-5}	1.00	0.85	0.89
	95th	1070.19	$< 10^{-6}$	4.90	4.67×10^{-2}	0.17	1	0.018	8.45×10^{-5}	1.00	0.92	0.85
White Matter	5th	6754.08	$< 10^{-6}$	33.39	$< 10^{-6}$	-13.87	$< 10^{-6}$	0.087	4.32×10^{-4}	1.00	0.75	0.66
	50th	1790.87	$< 10^{-6}$	15.40	$< 10^{-6}$	-5.74	$< 10^{-6}$	0.019	1.65×10^{-4}	1.00	0.93	0.78
	95th	728.82	3.93×10^{-2}	2.86	1	1.55	5.10×10^{-1}	0.007	2.58×10^{-5}	1.00	0.81	0.70
Lateral Ventricles	5th	170.82	$< 10^{-6}$	0.03	1	-0.02	1	0.060	1.13×10^{-5}	1.00	0.78	0.69
	50th	52.00	1.10×10^{-2}	-0.01	1	0.07	9.90×10^{-1}	0.012	-1.76×10^{-6}	1.00	0.85	0.86
	95th	112.79	4.53×10^{-2}	-0.11	1	0.26	4.54×10^{-1}	0.015	-1.51×10^{-5}	1.00	0.83	0.75
Cerebellum	5th	2470.67	$< 10^{-6}$	3.04	2.35×10^{-4}	-1.00	1.28×10^{-3}	0.680	8.33×10^{-4}	1.00	0.93	1.10
	50th	607.11	$< 10^{-6}$	-0.70	2.46×10^{-2}	0.30	7.07×10^{-3}	0.097	-1.12×10^{-4}	1.00	0.98	1.01
	95th	148.31	5.34×10^{-5}	-0.66	1.93×10^{-1}	0.34	2.79×10^{-2}	0.019	-8.29×10^{-5}	1.00	0.98	1.22
Basal Ganglia	5th	114.32	$< 10^{-6}$	-0.14	1	0.08	4.31×10^{-1}	0.031	-3.61×10^{-5}	1.00	0.67	0.65
	50th	-38.92	1.26×10^{-4}	-0.29	1.23×10^{-2}	0.11	1.20×10^{-2}	-0.008	-6.19×10^{-5}	1.00	0.97	0.93
	95th	-487.11	$< 10^{-6}$	0.51	1.57×10^{-1}	-0.05	1	-0.082	8.58×10^{-5}	1.00	0.79	0.78
Brainstem	5th	665.91	$< 10^{-6}$	0.28	5.10×10^{-1}	-0.13	2.51×10^{-1}	0.238	1.01×10^{-4}	1.00	0.78	0.75
	50th	163.37	$< 10^{-6}$	0.15	2.71×10^{-2}	-0.04	2.24×10^{-1}	0.044	4.09×10^{-5}	1.00	0.82	0.82
	95th	45.80	5.23×10^{-5}	0.55	2.44×10^{-6}	-0.08	1.51×10^{-1}	0.011	1.27×10^{-4}	1.00	0.84	0.77
Thalamus	5th	22.62	9.99×10^{-3}	-0.82	$< 10^{-6}$	0.24	$< 10^{-6}$	0.007	-2.67×10^{-4}	1.00	0.87	0.86
	50th	-17.55	1.44×10^{-2}	-0.10	4.39×10^{-1}	0.05	8.73×10^{-2}	-0.005	-2.67×10^{-5}	1.00	0.88	0.87
	95th	-94.26	$< 10^{-6}$	1.24	$< 10^{-6}$	-0.32	1.38×10^{-5}	-0.021	2.78×10^{-4}	1.00	0.87	0.87

Table S2: **Effect size from a LME model shows that the impact of QC on estimated centiles is consistent, large, and non-uniform**. Extended version of Table 2.

S7 Leave-dHCP-out experiment

Here, we reproduce the results of Figure 6 while leaving the dHCP cohort out of the experiment, to assess the robustness of our findings. The results are presented in Figures S7 and S8 and globally show a stronger effect of quality compared to the experiment where all data points are included (e.g. normalized E1 error on the order of 0.8 in the cerebellum compared to approximately 0.4 when including all data). Nevertheless, given the lower sample sizes in each group (n=343 in `All` and n=111 in `GroundTruth`), the results should be interpreted with caution, as variability due to sample sizes (especially for outer centiles) is more pronounced in this setting.

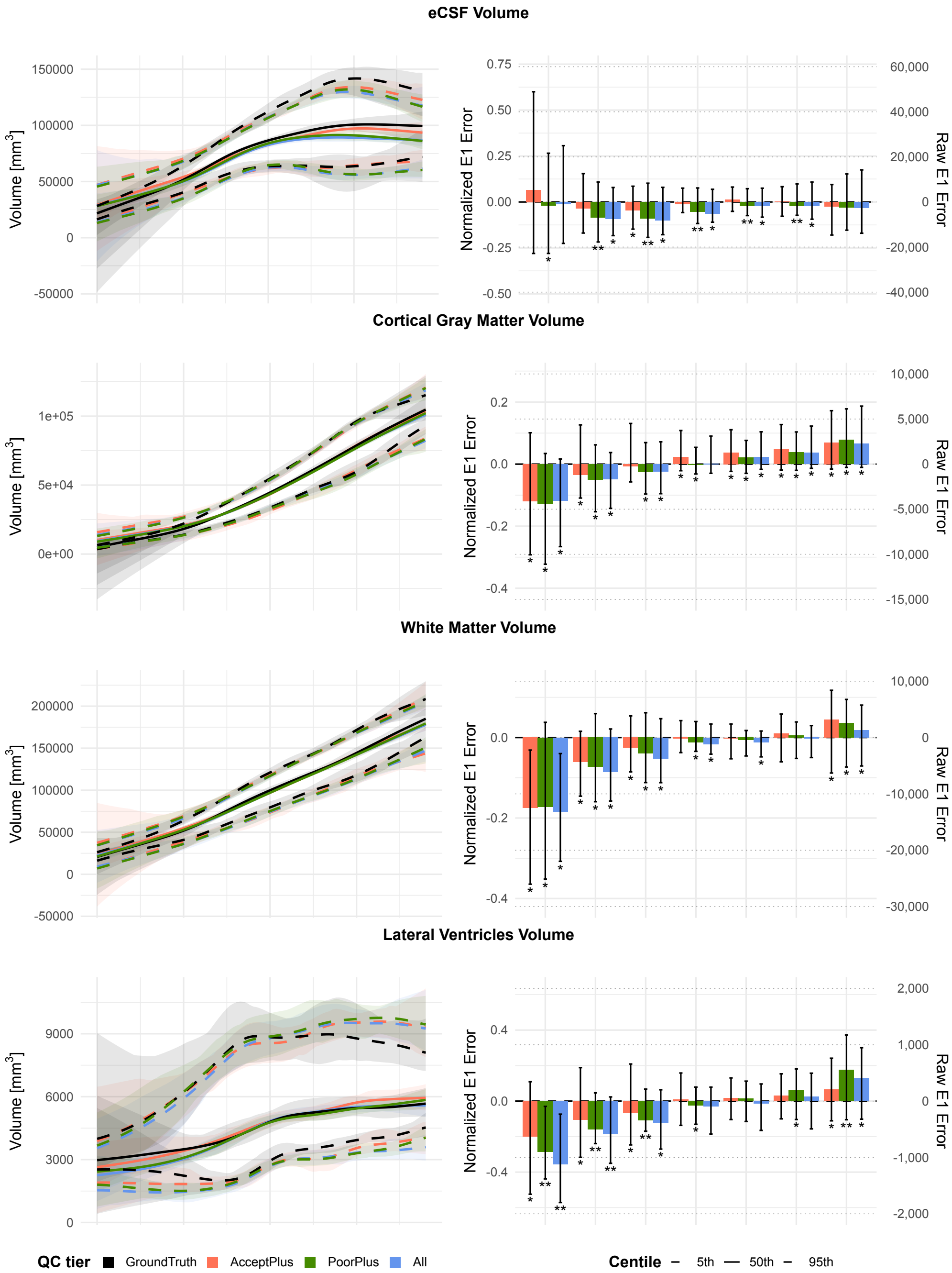

Figure S7: **Impact of data quality on normative curves in a leave-dHCP-out-experiment.** *First column:* Bootstrapped curves with 95% confidence intervals for the 5th, 50th and 95th centiles for data harmonized at each QC level. *Second column:* E_1 error distribution with 95% confidence intervals computed for 1st, 5th, 10th, 50th, 90th, 95th and 99th centiles for data harmonized at each QC level.

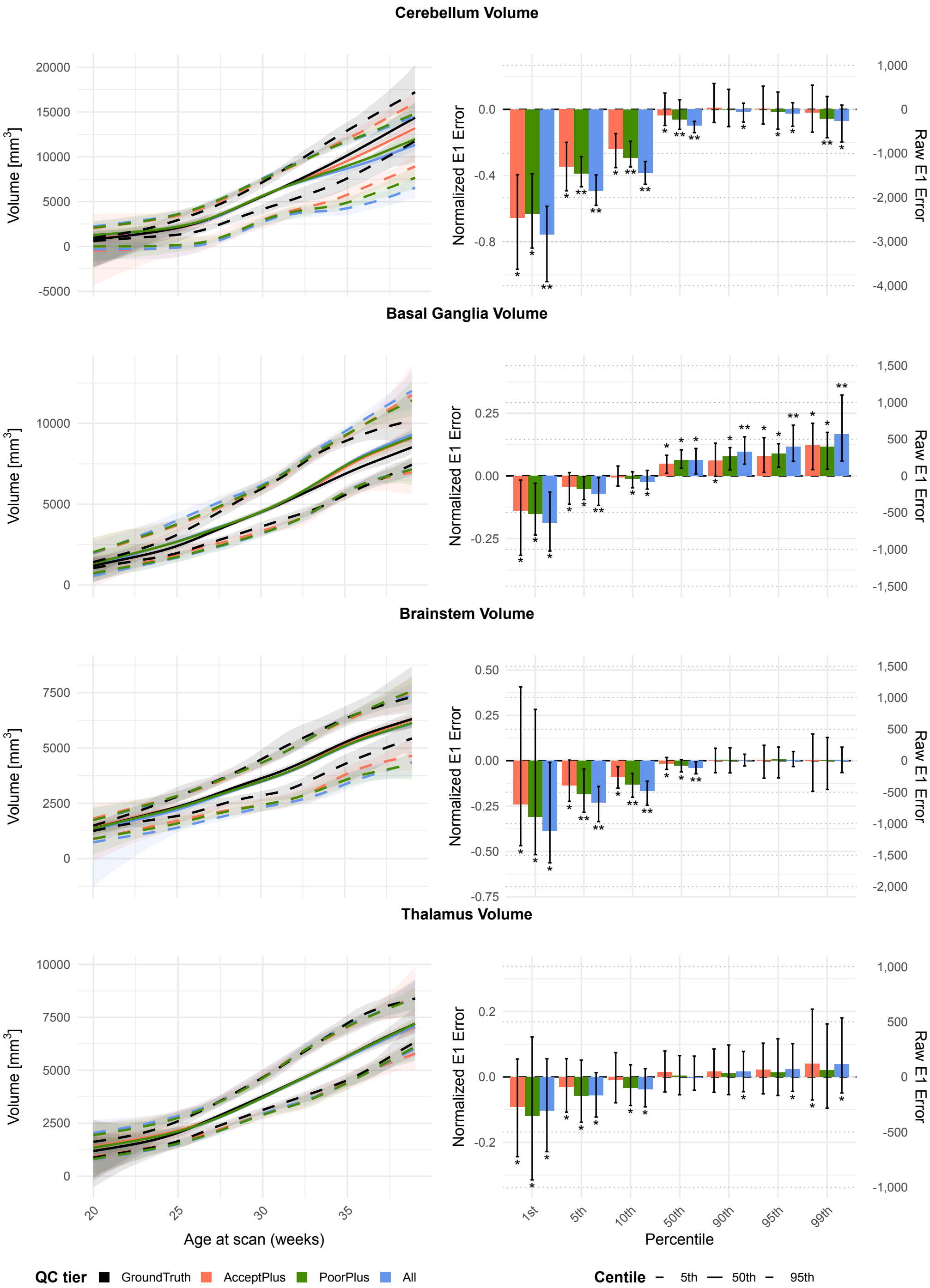

Figure S8: **Impact of data quality on normative curves in a leave-dHCP-out-experiment (cont'd).** *First column:* Bootstrapped curves with 95% confidence intervals for the 5th, 50th and 95th centiles for data harmonized at each QC level. *Second column:* E_1 error distribution with 95% confidence intervals computed for 1st, 5th, 10th, 50th, 90th, 95th and 99th centiles for data harmonized at each QC level.
